# Cytotoxic Helix-Rich Oligomer Formation by Melittin and Pancreatic Polypeptide

**DOI:** 10.1371/journal.pone.0120346

**Published:** 2015-03-24

**Authors:** Pradeep K. Singh, Dhiman Ghosh, Debanjan Tewari, Ganesh M. Mohite, Edmund Carvalho, Narendra Nath Jha, Reeba S. Jacob, Shruti Sahay, Rinti Banerjee, Amal K. Bera, Samir K. Maji

**Affiliations:** 1 Department of Biosciences and Bioengineering, IIT Bombay, Mumbai, Maharashtra, India; 2 Department of Biotechnology, IIT Madras, Chennai, Tamil Nadu, India; Children's Hospital of Pittsburgh, University of Pittsburgh Medical Center, UNITED STATES

## Abstract

Conversion of amyloid fibrils by many peptides/proteins involves cytotoxic helix-rich oligomers. However, their toxicity and biophysical studies remain largely unknown due to their highly dynamic nature. To address this, we chose two helical peptides (melittin, Mel and pancreatic polypeptide, PP) and studied their aggregation and toxicity. Mel converted its random coil structure to oligomeric helical structure upon binding to heparin; however, PP remained as helix after oligomerization. Interestingly, similar to Parkinson’s associated α-synuclein (AS) oligomers, Mel and PP also showed tinctorial properties, higher hydrophobic surface exposure, cellular toxicity and membrane pore formation after oligomerization in the presence of heparin. We suggest that helix-rich oligomers with exposed hydrophobic surface are highly cytotoxic to cells irrespective of their disease association. Moreover as Mel and PP (in the presence of heparin) instantly self-assemble into stable helix-rich amyloidogenic oligomers; they could be represented as models for understanding the biophysical and cytotoxic properties of helix-rich intermediates in detail.

## Introduction

Self-assembly process of proteins/peptides into oligomers and amyloid fibrils is important to study because this process creates many human diseases such as Alzheimer's disease (AD) and Parkinson's disease (PD) [1,2]. Although amyloid fibrils were thought to be the toxic species responsible for cell death, which occurs in amyloid diseases, recent studies, however, have shown that soluble oligomers are more cytotoxic than mature fibrils [3,4]. In many neurodegenerative disorders, direct evidences were achieved to show that soluble oligomers are the most plausible cytotoxins responsible for diseases [4,5]. For example, using oligomer-forming mutant, recently it has been shown that AS oligomers are more cytotoxic compared to AS fibrils *in vivo* [4,6]. Similarly, in AD, different sized cytotoxic Aβ oligomers have been also discovered [5,7–12], many of which showed cytotoxicity and cell death in *vitro* and *in vivo*. Interestingly, proteins/peptides, which do not have any disease connections, can also form highly cytotoxic amyloid oligomers [13,14]. For example, the amino-terminal domain of *E*.*coli* HypF protein, SH3 domain of bovine-phosphatidyl-inositol-3’-kinase and hen egg white lysozyme protein can assemble into inherently cytotoxic amyloid oligomers [13,14]. These findings suggest that cytotoxicity could be a generic property of many protein/peptide oligomers.

During amyloid aggregation, proteins/peptides form partially folded intermediates, soluble oligomers and subsequently assemble into β-sheet rich amyloid fibrils [15]. Previously, many studies have shown that during aggregation, natively unstructured proteins/peptides form helix-rich intermediates as penultimate precursors of β-sheet fibrils [16–18]. These helical oligomers are proposed to be cytotoxic and, therefore, are promising drug target in the treatment of many amyloid-related disorders. For example, it has been shown that helix-rich oligomers of islet amyloid polypeptide (IAPP) (associated with type II diabetes) are highly cytotoxic and they were able to induce apoptosis in pancreatic β cells [19]. In the previous studies, direct evidences for helical intermediate formation are shown for Aβ and IAPP associated with Alzheimer's and Type II diabetes, respectively [17,18]. However, reports of helical oligomers for other disease associated proteins are not very much explored and shown to appear only under certain experimental conditions [17]. For example, it has been shown that PD associated unstructured α-synuclein (AS) can form ordered helical oligomers in the membrane mimicking condition [20]. Furthermore, the short-life time of these helical oligomers does not make them amenable for studying their detailed biophysical characterization and their mode of toxicity [21]. Therefore, designing stable helix-rich oligomers will be helpful in elucidating their toxic mechanism and biophysical characterization.

To elucidate the mode of toxicity and biophysical characterization of helical oligomers in general, we chose two unrelated peptides, melittin (Mel) and pancreatic polypeptide (PP) (S1 Fig.) and studied their aggregation and toxicity in presence of a glycosaminoglycan, heparin. Heparin was used in this study as it is known to induce amyloid aggregation in many peptides/proteins [22]. Heparin was chosen for one more reason that glycosaminoglycans are cell surface molecules [23] and known to interact with proteins/peptides, thereby modulating their structure-function relationship on cell interface [24,25].

Mel is a peptide (26-residue) component of bee venom and is known to possess cytolytic as well as antimicrobial properties [26]. Mel is shown to acquire an unstructured conformation in an aqueous environment; however, has been shown to assemble into helix-rich tetramers upon insertion into the membrane and also in other designed experimental conditions [27]. Besides its tetramer formation tendency, the amyloidogenic nature of Mel is not known and characterization of its higher order assembly is also poorly understood. In contrast to Mel, PP (36-residue) is a peptide hormone, which is secreted by PP/γ cells of the islet of Langerhans [28] and not known to possess any toxic property. Additionally, PP is a well-known pancreatic hormone with stable helical fold called PP-fold [29]. Although other pancreatic peptide hormones (insulin, somatostatin and glucagon) are shown to form amyloid aggregates [22,30,31], the amyloidogenecity of PP is not yet reported.

In this study, we found that Mel and PP formed cytotoxic helical oligomers of globular morphology in the presence of heparin. We compared the toxic mechanism of these oligomers with PD associated AS oligomers [32] and found that similar to AS oligomers, Mel and PP oligomers also possess exposed hydrophobic surfaces and channel formation activity in artificial bilayer lipid membrane (BLM). These helical oligomers of Mel and PP (formed in the presence of heparin) are stable and, therefore, could be used as model oligomers for elucidating toxicity, as well as biophysical properties of amyloidogenic helix-rich oligomers.

## Materials and Methods

### Chemicals and reagents

The peptides (Mel and PP) were purchased from BACHEM (Switzerland) with the highest purity available. Wild-type α-synuclein plasmid construct (AS-pRK172) was gifted by Prof. Roland Riek, ETH Zurich, Switzerland. All chemicals and reagents, unless otherwise specified, were purchased from Sigma, USA.

### Peptide oligomerization

To study the peptide oligomerization, Mel and PP were dissolved in 0.5 ml of 5% D-mannitol, 0.01% sodium azide, and pH 5.5 at a concentration of 2 mg/ml in 1.5 ml eppendorf tubes. The eppendorf tubes containing peptide solutions were placed into an EchoTherm model RT11 rotating mixture (Torrey Pines Scientific, USA) and rotated at 50 rpm inside a 37°C incubator. Similarly, to study the peptide oligomerization in the presence of heparin, PP and Mel were dissolved in 0.5 ml of 5% D-mannitol, 0.01% sodium azide, pH 5.5 at a concentration of 2 mg/ml in presence of 400 μM low molecular weight (LMW) heparin (MW 5 kDa, CalBioChem) in 1.5 ml eppendorf tubes and were incubated as described above. For SDS induced aggregation study, 25 μM of Mel in Gly-NaOH buffer (pH 9.2) was incubated with and without 2.5 mM SDS at 37°C.

The α-synuclein (AS) protein was expressed in *E*. *coli* (BL21) cells and purified as previously described by Volles and Lansbury [33] with slight modification [34,35]. To isolate the preformed AS oligomers, lyophilized protein (10 mg/ml) was solubilized in PBS (pH 7.4) as described before [34]. The protein solution was then centrifuged (18,000 x g, 4°C, 30 min), to remove any aggregated fibrillar species and the clear supernatant was injected in size exclusion chromatography (SEC) column (Superdex 200 10/300 GL). The elution was performed in the same buffer at 4°C with AKTA purifier (GE Healthcare) at a flow rate of 0.4 ml/min. The fraction close to the void volume (8.0 ml) contains oligomeric species [34], which were collected and used for further biophysical characterization and toxicity measurement. Monomeric fraction (close to 15 ml of elution) was also collected and used as control.

For isolating pure oligomers of Mel and PP, we used 10 KDa molecular weight cut-off (MWCO) Amicon Ultra (0.5 ml) centrifugal filters (Merck Millipore, Germany). These centrifugal filters were used as per manufacturer’s instructions. Two weeks incubated Mel and PP (in the presence of heparin) samples were used for isolating the pure oligomers by centrifugation method. Since monomeric Mel (2846.50 Da) and PP (4181.77 Da) have molecular weights below 10 KDa, during centrifugation, the upper fraction of filter retained mostly oligomeric species (retentate) and the flow-through fractions were mostly comprised of monomeric entities. The isolated retentates of Mel and PP were further used for biophysical characterization.

### Circular dichroism (CD) spectroscopy

For the secondary structural analysis, CD spectroscopy was performed. For this study, 15 μl of each peptide solution (2 mg/ml) was diluted to 200 μl in 5% D-mannitol, 0.01% sodium azide, pH 5.5. The sample was then placed into a 0.1 cm path-length quartz cell (Hellma, Forest Hills, NY) and CD spectra were acquired at 25°C using JASCO J-810 CD spectropolarimeter. Spectra were recorded in the range of 198–260 nm. AS (monomers and oligomers) isolated from SEC were also used for CD spectroscopy. Three independent experiments were performed with each sample. CD spectra of Mel (25 μM) in the presence of SDS (2.5 mM) and liposomes were also recorded similarly. Smoothing of raw data and subtraction of buffer spectra were done as per manufacturer’s instructions.

### Fourier transform infrared spectroscopy (FTIR)

Secondary structural analysis of two weeks incubated peptide samples (in the presence and absence of heparin) were carried out using FTIR spectroscopy. For this study, the samples were prepared as described before [34]. FTIR spectra were acquired in the spectral range of 1800–1500 cm^-1^ with Bruker Vertex-80 instrument equipped with DTGS detector [34]. For each spectrum, 32 scans at the resolution of 4 cm^-1^ were recorded and the resultant absorption spectra were deconvoluted and curve fitted in the amide-I region (1700–1600 cm^-1^) as per manufacturer’s instructions.

### Atomic force microscopy (AFM)

To evaluate the morphology of oligomers, AFM analysis was performed. For this study, the samples were diluted to a final concentration of 10 μM (in double distilled water) and spotted on a freshly cleaved mica sheet for 1 min at room temperature (RT). The mica sheets were then washed with double distilled water and dried in a vacuum desiccator. The imaging was done using Veeco Nanoscope IV Multimode AFM in tapping mode with etched silicon cantilever. Minimum five different areas of three independent samples were scanned with a scan rate of 1.5 Hz.

### Electron microscopy (EM)

To study the morphology of oligomers under an electron microscope, samples were diluted with double distilled water to reach the peptide concentration of 50 μM. The diluted solutions were spotted on a glow-discharged, carbon-coated formvar grid (Electron Microscopy Sciences, Fort Washington, PA), incubated for 5 min on the grid and washed with double distilled water two times, and finally stained with 1% (w/v) aqueous uranyl formate solution. The air-dried EM grids were used for the imaging. EM analysis was performed using the electron microscope (FEI Tecnai G2 12) at 120 kV with nominal magnifications in the range of 26,000 to 60,000. Images were recorded digitally using SIS Megaview III imaging system. At least two independent experiments were carried out for each sample.

### Dynamic light scattering (DLS) experiment

DLS experiment was performed using DynaPro-MS800 instrument (Protein Solutions Inc.). It monitors the scattered light at 90° relative to the excitation. A 50 μl of 2 mg/ml solutions of two weeks incubated peptides (in the presence and absence of heparin) was used for size analysis using DLS. Water, buffer alone and buffer with heparin were used as controls. The preformed AS oligomers (isolated from SEC) were also used for size analysis. For each sample, at least 30 measurements each with 5-s duration were performed. Two sets of experiments were performed independently and raw data were processed with the software provided by the manufacturer.

### Thioflavin T (ThT) binding assay

To analyze the amyloidogenic nature (tinctorial properties) of peptide oligomers, ThT binding assay was performed. For this study, a 5 l aliquot of each sample was diluted to 200 μl in 5% D-mannitol and 2 μl of 1 mM ThT was added to it. The samples were then excited at 450 nm and emission spectra were recorded in the range of 460–500 nm using Horiba-JY (Fluoromax 4) spectrofluorometer. Three independent experiments were performed for each sample and the emission intensity values at 480 nm were plotted. The slit widths of 5 nm were used for both excitation and emission. For measuring the ThT binding of Mel (incubated with SDS), 4 μl ThT was added to 200 μl solution containing 25 μM of Mel. ThT binding was performed immediately after addition of SDS (d0) and also after 5 days of incubation (d5).

### Congo red (CR) binding assay

To evaluate the amyloidogenic properties of Mel and PP oligomers, CR binding assay was performed [36]. For this study, 20 μl aliquot of incubated sample was mixed with 150 μl of PBS buffer (containing 10% ethanol). Then 30 μl of 100 μM CR solution (prepared in PBS containing 10% ethanol) was added to the samples and incubated for 10 min in dark at RT. After this, the CR absorbance was measured in the range of 300 to 700 nm using JASCO V-650 spectrophotometer. Similarly, CR absorbance of AS monomers, AS oligomers, and CR alone were recorded as controls. Three independent experiments were performed for each sample and the absorbance values at 510 nm were plotted.

### Prediction of oligomerization propensity

The intrinsic oligomerization ability of Mel and PP was calculated (at pH 5.5) using Zyggregator software [37] with default parameters.

### Dot blot assay

Dot blot assays were performed with oligomer specific A11 [5] and fibril specific OC antibody [38]. For this study, two weeks incubated peptide samples (in presence and absence of heparin) and AS oligomers isolated from SEC were used. AS monomers (isolated from SEC) and preformed AS fibrils were also used as controls. For this, 5 μl of each sample was spotted on the nitrocellulose membrane (Immobilon-NC, Millipore) and then air-dried for 10 min at RT. After the air-drying, two subsequent washes (2 x 8 min) were performed with PBST (137 mM NaCl, 2.7 mM KCl, 10 mM Na_2_HPO_4_, 2mM KH_2_PO_4_, and 0.1% tween 20). The blots were then blocked with 5% non-fat milk powder (Himedia, India) in PBST for 1 h at RT and then incubated with oligomer specific A11 antibody (dilution-1: 500, AHB0052, Invitrogen). Another blot was used for fibrils specific OC antibody (dilution-1: 600, AB2286, Millipore). The incubations were performed at 4°C. After overnight incubation, blots were washed twice (2x8 min) with PBST and again incubated with horseradish peroxidase (HRP) conjugated secondary antibody (dilution-1: 1000, Cat. 401253, Calbiochem). Finally, three subsequent washes were performed with TBST (50 mM Tris, 150 mM NaCl, and 0.1% tween 20) and the blots were developed with chemiluminescent substrate (West Pico, Pierce Thermo Scientific, USA).

### Cell morphology analysis

To evaluate the cellular toxicity of oligomers, morphology analysis of oligomers treated and untreated SH-SY5Y cells were performed. In brief, cells were seeded onto sterile coverslips at a density of 10,000 cells per well in 24 well cell culture dish and incubated for 24 h. After incubation, media were discarded. Fresh media with peptide samples were added to the cells such that the final peptide concentration was 10 μM. As a control, a similar volume of D-mannitol was also diluted in media and added to cells. The cells were further incubated in a 5% CO_2_ humidified environment at 37°C. After 30 h of incubation, cell morphology was directly visualized under phase contrast microscope (Olympus IX-51).

### Lactate dehydrogenase release (LDH) assay

To quantify the cellular toxicity of oligomers, lactate dehydrogenase (LDH) release assay [39] was performed using SH-SY5Y neuronal cell line. SH-SY5Y cells were cultured in Dulbecco's Modified Eagle Medium (DMEM) (Himedia, India) supplemented with 10% FBS (Invitrogen, USA), 100 units/ml penicillin and 100 μg/ml streptomycin in a 5% CO_2_ humidified environment at 37°C. For LDH assay, cells were seeded in 96-well plates in 100 μl medium at a cell density of ~10,000 per well and incubated for 24 h. After incubation, cell culture medium was replaced with fresh medium containing different concentrations of oligomers (2.5 μM, 5.0 μM and 10 μM) and cells were incubated for 30 h. AS monomers and freshly dissolved Mel were also used in this experiment. After incubation, LDH assay was performed using LDH toxicological kit (TOX-7, Sigma, USA), according to the manufacturer’s instructions. For positive control (100% cell death), 0.5% TritonX-100 was used and only 5% D-mannitol alone in cell culture media was used as a negative control. The percentage of cell death was calculated by considering 100% cell death, when cells were treated with 0.5% TritonX-100.

### Liposome preparation

Liposomes were prepared using 1,2-dipalmitoyl-sn-glycero-3-phosphocholine (DPPC) and 1,2-dipalmitoyl-sn-glycero-3-phospho- (1'-rac-glycerol) (sodium salt) (Lipoid GmbH, Germany) (DPPG). Chloroform-containing lipid solutions were dried in a rotary vacuum evaporator in order to obtain a thin film. Residual chloroform was removed under vacuum. For cryo-SEM studies, the lipid film was hydrated in phosphate buffer saline (PBS), pH 7.4 for 30 min at 45°C and then used. For the calcein release assay, the dried lipid film resulting from evaporation was resuspended in 25 mM aqueous calcein dye (Sigma, USA), which was prepared in 2N NaOH and the pH was adjusted to 7.4 in PBS (pH 7.4). The resuspended solution, at a final lipid concentration of 4 mg/ml, was incubated for 30 min at 45°C with 100 rpm rotation to allow the vesicle formation. After 30 min, the lipid suspensions were sonicated at 40 KHz, 40% amplitude for 3 min to form small unilamellar vesicles (100–200 nm diameters). To remove the excess calcein, the solution was centrifuged at 4°C for 30 min with a speed of 18,000 x g and the supernatant was discarded. The pellet was suspended gently in PBS (pH 7.4) and the centrifugation was repeated thrice. The calcein-loaded liposome was diluted to 100 fold in PBS and the calcein fluorescence was measured before and after adding 0.5% Triton X-100. After treatment with Triton X-100, high increase in calcein fluorescence (excitation at 490 nm, emission in the range of 500–600 nm) was obtained, however, very minimal calcein fluorescence (~10%, background fluorescence) was observed in the absence of Triton X-100. The liposomes were used immediately for the study.

### Liposome damage study

To visualize the oligomer mediated liposome damage; freshly prepared liposomes (~400–700 nm diameters) were used in the study. The liposomes were diluted to 100 fold in PBS (pH 7.4) and incubated with 10 μM oligomers (Mel and PP) in a reaction volume of 50 μl at RT for 30 min. After incubation, morphology of treated and untreated liposomes was visualized using cryo-FEG SEM (JSM-7800F-thermal field emission scanning electron microscope, JEOL).

### Nile Red (NR) binding assay

NR is a hydrophobic dye that is frequently used to measure the extent of hydrophobic surface exposure of proteins/peptides [35,40]. Preformed AS oligomers and two weeks incubated Mel/PP samples were diluted in 200 l of 5% D-mannitol such that the final concentration became 10 μM in which 0.2 l of 1 mM NR (prepared in DMSO) was added to the solution. The mixture was incubated for 5 min in dark at RT. The NR fluorescence was recorded using Horiba-JY (Fluoromax 4) with excitation at 550 nm and emission from 565–750 nm. The excitation and emission slit widths were 2 nm and 5 nm, respectively. For controls, fluorescence spectra of NR alone and NR in presence of AS monomers (isolated from SEC) were also recorded under similar conditions.

### Planar bilayer recordings

Artificial bilayer lipid membrane (BLM) was constructed from 1, 2-diphytamoyl-*sn*-glycero-3-phosphocholine (DPhPC; Avanti Polar Lipids, Alabaster. AL). DPhPC, dissolved in n-decane (20 mg/ml) was painted in a small aperture (150 mM diameter), partitioning two aqueous chambers in a Delrin cuvette (Warner Instrument, USA). The cis and trans chambers were filled with symmetrical solution of 1 M KCl, 5 M MgCl_2_ and 10 mM HEPES (pH 7.4). The cis chamber was held at virtual ground and the trans chamber was connected to the head-stage of amplifier (Axopatch 200B, Molecular Probes, USA). Mel and PP (incubated with and without heparin for two weeks) were added (1μM) to the cis and stirred for 5–10 min. AS oligomers and monomers (isolated from SEC), were also included in the study. Channel activity was monitored at different voltages. Data was filtered at 1 kHz (low pass) and digitized at 5 kHz using amplifier Axopatch 200B (Molecular Devices, USA). The pClamp software (version 9, Molecular Devices) was used for data acquisition and analysis. Additional analysis was done using Sigma Plot 11. Single channel conductance was calculated from all point histogram.

### Calcein release assay

To study the dye leakage ability of oligomers, calcein release assay was performed using calcein-loaded liposomes. The freshly prepared calcein-loaded liposomes were 100 fold diluted in PBS (pH 7.4) before starting the experiment. The oligomers were added to these diluted liposomes at a final concentration of 10 μM and in a reaction volume of 150 μl. Peptide samples incubated in the absence of heparin and AS monomers isolated from SEC were also used as controls. To achieve 100% calcein release, 0.5% Triton X-100 was used as a positive control. The reaction was started in a clear bottom 96 well fluorescence plate (Sigma, USA) and the time-dependent fluorescence intensity (at 520 nm) was recorded (excitation at 495 nm) at 25°C using spectraMax M2e microplate reader (Molecular Devices, USA).

## Results

### Mel and PP form helix-rich globular oligomers

**Fig 1:**
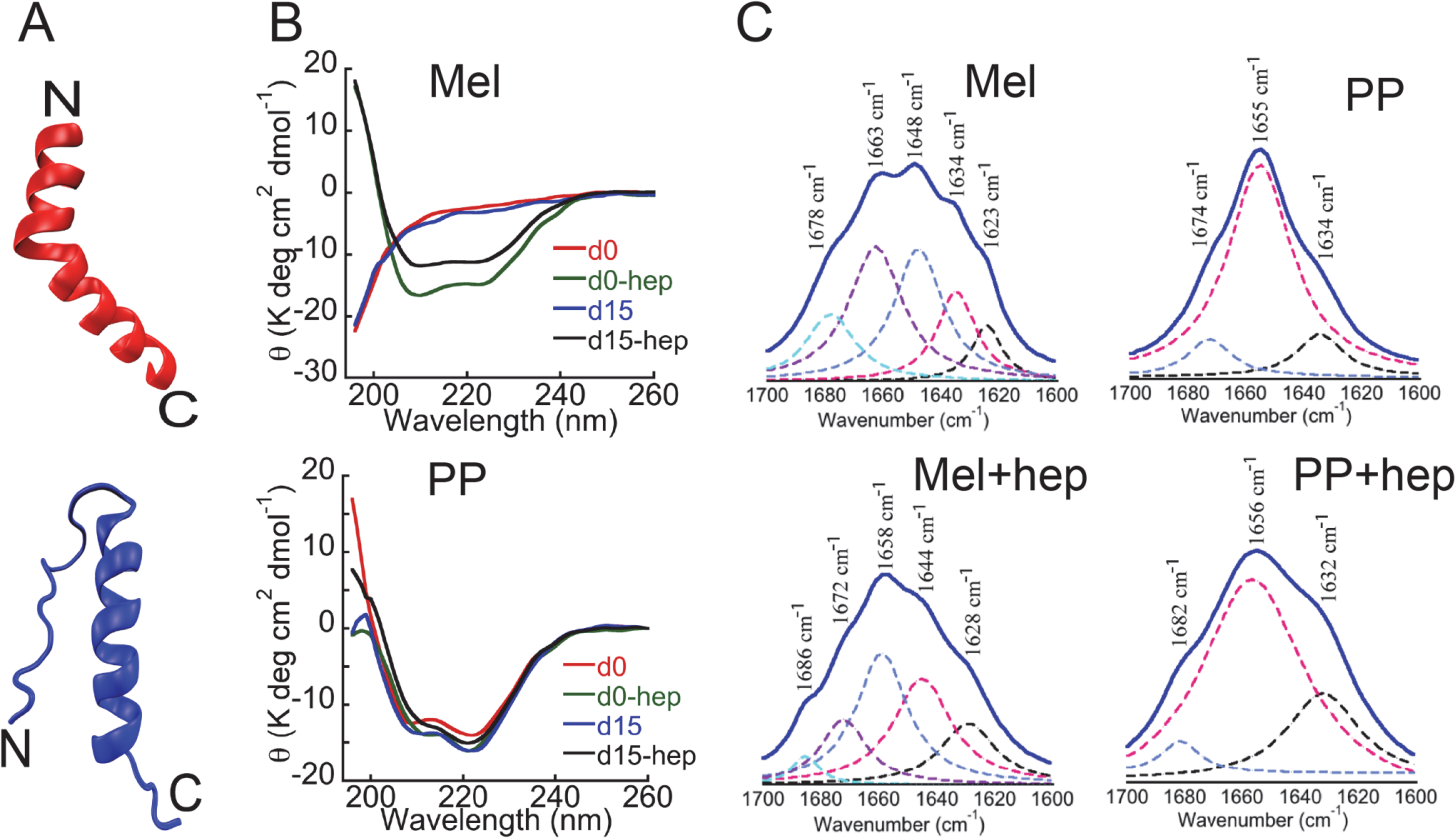
Structural characterization of Mel and PP. **(A)** Structural model of Mel (red, PDB ID: 2MLT) and PP (blue, bovine PDB ID: 1BBA). **(B)** CD spectra of Mel and PP at day 0 (d0) and after 15 days (d15) in presence and absence of heparin. After the addition of heparin and subsequent incubation for two weeks, the secondary structure of PP remained mostly unchanged (helical). **(C)** FTIR spectra of two weeks incubated PP and Mel (in the absence and presence of heparin). Y-axis represents the absorbance (AU) and X-axis represents the wavenumber (cm^-1^). Wavenumbers corresponding to the maximum absorbance are represented with arrow marks. Consistent with CD data, FTIR study also showed that in the presence of heparin, unstructured Mel transformed into helical conformation, whereas PP remained mostly helical both in presence and absence of heparin after incubation.

Both, Mel and PP possess helical propensity as shown in Fig. 1A. For studying the oligomerization, Mel and PP were dissolved in 5% D-mannitol at a concentration of 2 mg/ml (with and without 400 μM heparin) and incubated at 37°C with slight rotation. To evaluate the secondary structure of Mel and PP (in presence and absence of heparin), CD spectroscopy was performed before and after two weeks of incubation. Immediately after dissolution, Mel showed the mostly unstructured conformation as evident from single negative minima near 198 nm in far-UV CD spectroscopy (Fig. 1B). However, Far-UV CD spectrum of PP showed two negative minima; one at ~ 222 nm and another at ~208 nm, respectively, characteristics of helix-rich conformation. Interestingly, PP did not change its helical conformation even after addition of the heparin, suggesting that heparin might not be able to induce further structural transition in PP (Fig. 1B). However, when heparin was added to Mel peptide, it immediately transformed into helical conformation, as evident by two negative minima near 222 nm and 208 nm, respectively in its far-UV CD spectrum (Fig. 1B).

The CD data suggest that unlike PP, heparin interaction to Mel peptide induced a drastic structural rearrangement. Further structural analysis of these samples after two weeks showed that Mel and PP (in the presence of heparin) retained their helicity during the course of incubation (Fig. 1B). The CD data thus suggest that helical conformations of Mel and PP were fairly stable and resisted any subsequent structural transition. PP (incubated in the absence of heparin) also showed helical conformation, however, Mel peptide, which was incubated in the absence of heparin, remained mostly unstructured (Fig. 1B). Consistent with CD data, the FTIR spectroscopy also revealed that PP samples incubated in absence and presence of heparin were of mainly helical conformation as characterized by the absorbance maxima at 1655 cm^-1^ and 1656 cm^-1^, respectively (Fig. 1C). However, Mel showed large conformational transition from RC (1648 cm^-1^) to helix (1658 cm^-1^) due to the addition of heparin (Fig. 1C). The CD and FTIR data of Mel, thus suggest that even though Mel has helical propensity, it alone cannot undergo structural transition and requires either helix-favoring condition or any additive like heparin.

**Fig 2:**
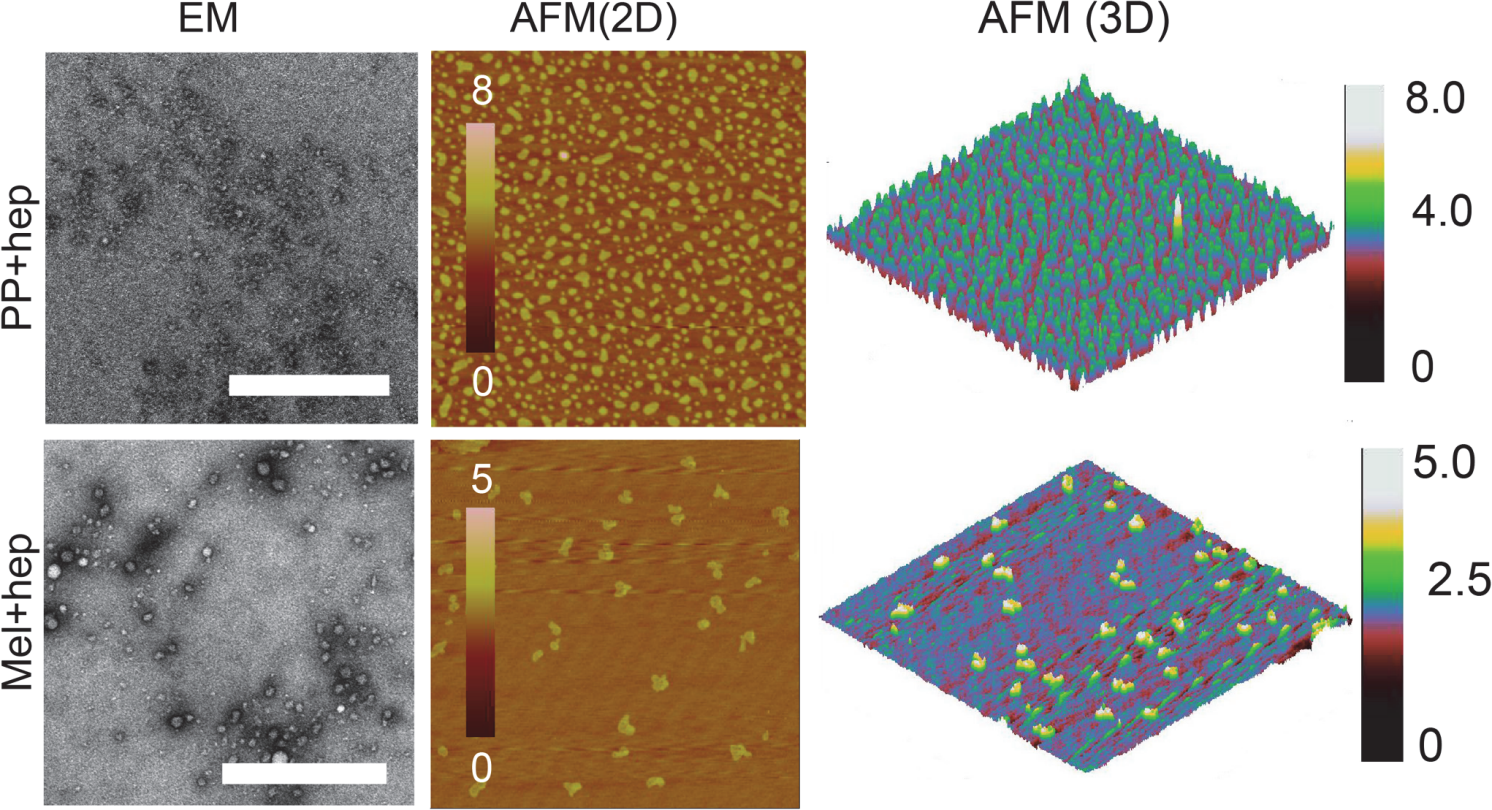
Morphological characterization of Mel and PP oligomers. EM and AFM analysis were performed to visualize the morphology of two weeks incubated Mel and PP (in the presence of heparin). EM (left panel) and AFM (middle panel) images showing oligomer formation in the presence of heparin. The right panel shows 3D AFM height images of oligomer. Scale bars for EM images are 500 nm. Height scales for AFM images are also shown.

Further, we analyzed the morphology of PP and Mel incubated both in presence and absence of heparin. AFM analysis of Mel sample (in the presence of heparin) showed globular oligomers (S2 Fig.), however, these oligomeric species were mostly absent in Mel alone sample (S2 Fig.). This data suggests that structural transition in Mel (in the presence of heparin) might have initiated oligomerization. We also examined the morphology of PP (in presence and absence of heparin) and we found that heparin also induced instant oligomerization in PP (S2 Fig.). Further morphology analysis of two weeks incubated samples by EM and AFM showed that the size of oligomers increased during incubation; however, they remained mostly globular in morphology (Fig. 2). Interestingly, the microscopy data revealed that Mel formed large oligomers, whereas PP showed relatively small oligomers, in the presence of heparin (Fig. 2). PP incubated in the absence of heparin did not show any globular oligomeric species, however, it showed some amorphous like structure in EM (S3 Fig.), suggesting that heparin is required for these oligomeric assemblies. However, Mel sample, which was incubated in the absence of heparin also showed oligomeric species in EM and AFM but smaller than Mel oligomers formed in the presence of heparin (S3 Fig.). This data suggests that Mel has propensity to self-assemble, however, this process can be accelerated in the presence of heparin. It is interesting to note that, both Mel and PP possess stretches of basic amino acids (I^20^-K^21^-R^22^-K^23^-R^24^-Q^25^ for Mel and R^33^-P^34^-R^35^ for PP) (S1 Fig.), which might be responsible for interaction with anionic polymer, heparin [41,42].

**Fig 3:**
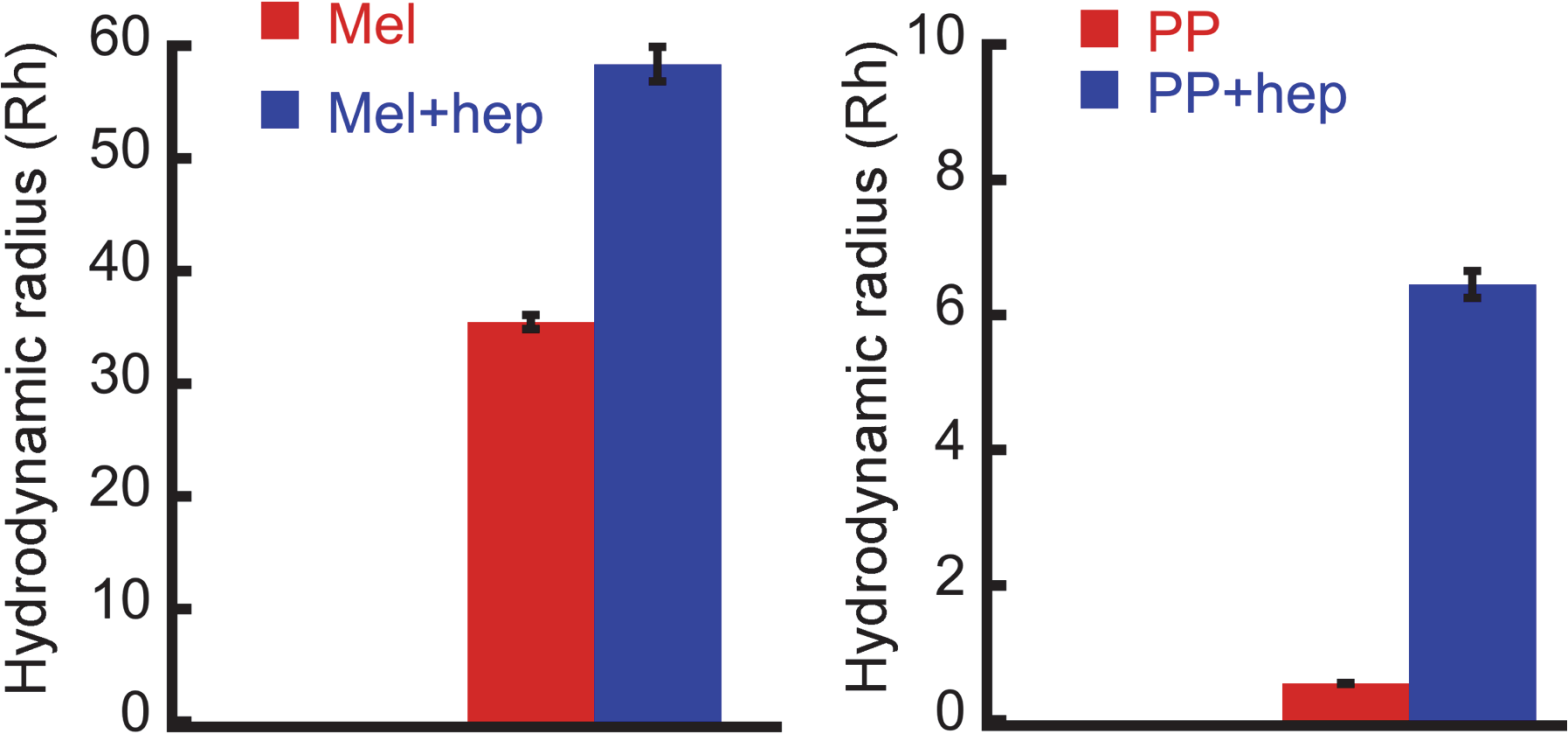
Hydrodynamic radius of oligomers. Dynamic light scattering (DLS) was performed to obtain the hydrodynamic radius (Rh) of peptide samples incubated for two weeks in presence and absence of heparin. The Rh values of peptides incubated in the presence of heparin increased considerably.

To further characterize the oligomers size in solution, DLS experiment was performed. The two weeks incubated peptide samples (in the presence and absence of heparin) were used for DLS experiment and their hydrodynamic radii were measured (Fig. 3). The DLS analysis revealed that the Mel peptide, which was incubated in the absence of heparin has average hydrodynamic radius (Rh) of 35.5±0.6 nm. However, Mel peptide, which was incubated in the presence of heparin, has average Rh of 58.4±1.6 nm. Furthermore, PP sample, which was incubated in the presence of heparin, has average Rh of 6.5±0.2 nm. However, the Rh value of PP sample incubated in the absence of heparin was 0.55±0.01 nm (Fig. 3), suggesting that the addition of heparin caused oligomerization of PP. The DLS data thus correlate well with AFM and EM results and collectively suggest that heparin accelerated the oligomerization of Mel and promoted its assembly into bigger oligomers. However, PP oligomerized in the presence of heparin only and showed lesser Rh compared to Mel oligomers.

### Tinctorial properties of Mel and PP oligomers formed in the presence of heparin

**Fig 4:**
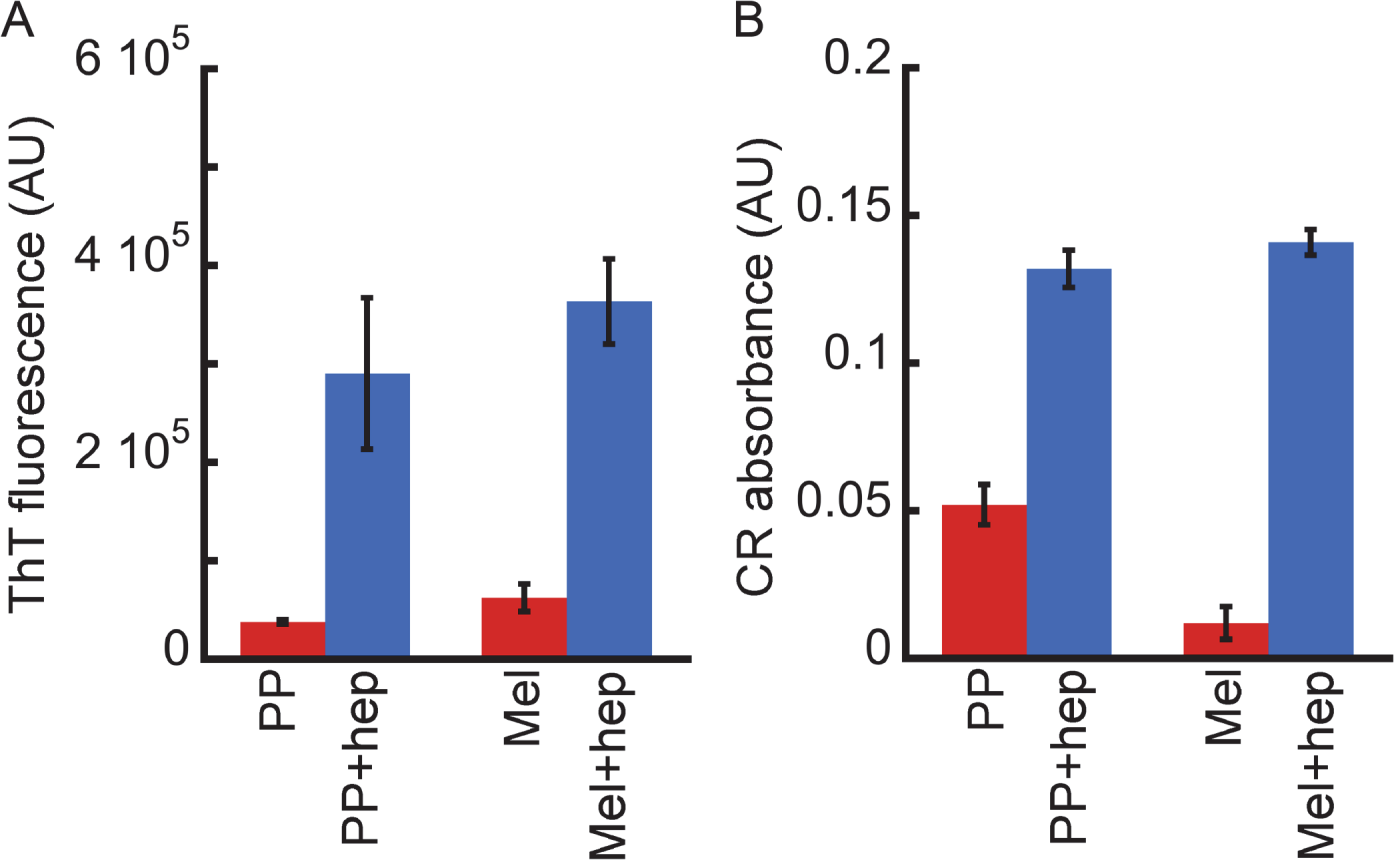
Tinctorial properties of Mel and PP oligomers. **(A)** ThT binding assay and **(B)** CR binding assay of two weeks incubated Mel and PP (in the presence and absence of heparin). ThT and CR binding showing that both Mel and PP oligomers, which were formed in the presence of heparin, bind moderately with these dyes.

Although both peptides (Mel and PP) are not involved in amyloid diseases, we checked whether Mel and PP oligomers, which were formed in presence of heparin, bind to any amyloid specific dye such as ThT and CR. In this context, it has been recently shown that many peptides/proteins, which are not associated with any neurological disorder, also form cytotoxic oligomers, which show tinctorial properties of amyloids [13,14]. When bound to amyloids or amyloidogenic oligomers, ThT gives a significantly high fluorescence emission signal at 480 nm when excited at 450 nm [43]. Similarly the molar absorptivity of CR (at ~540 nm) increases after binding with amyloid oligomers/fibrils [36,44,45]. We found that Mel and PP oligomers, which were formed in the presence of heparin, moderately bound to ThT (Fig. 4A) and CR dye (Fig. 4B), suggesting their amyloidogenic nature. PP and Mel, which were incubated in the absence of heparin, did not show significant ThT and CR binding, suggesting that heparin has induced amyloidogenic oligomer formation.

**Fig 5:**
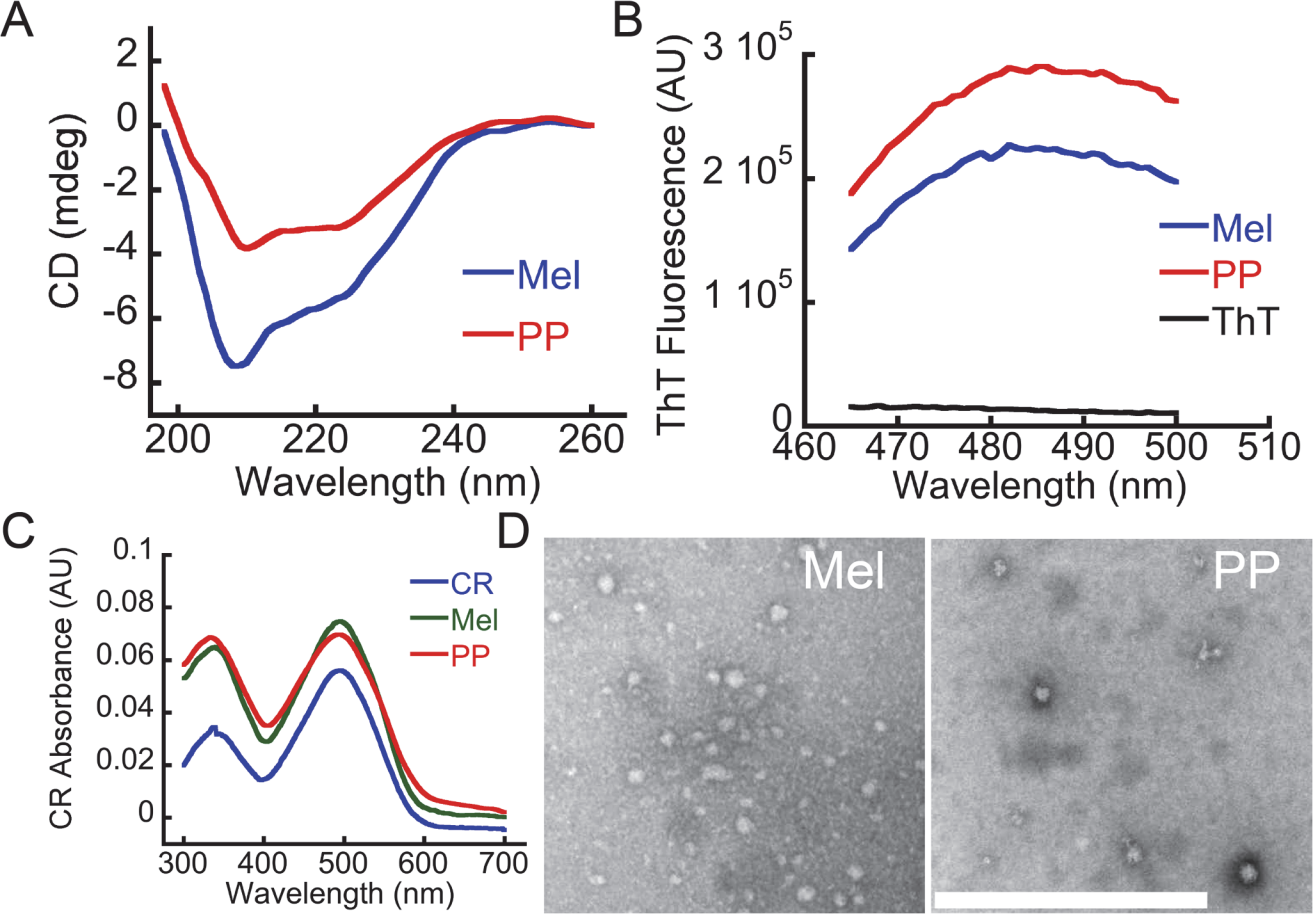
Biophysical characterization of isolated Mel and PP oligomers. **(A)** CD spectroscopy of isolated oligomers of Mel and PP in the presence of heparin. Both oligomers showed helical conformation in CD. **(B)** ThT fluorescence of the isolated Mel and PP oligomers showing moderate ThT binding. **(C)** CR binding of the isolated Mel and PP oligomers. **(D)** EM images showing large globular oligomeric morphology of the isolated Mel and PP oligomers formed in the presence of heparin. Scale bar is 500 nm.

We also isolated the pure oligomers of Mel and PP (using 10 KDa MWCO centrifugal filters) for their further structural and biophysical characterization. Consistent with data obtained prior to isolation, the isolated pure oligomers of Mel and PP also showed helix-rich conformation (Fig. 5A), and moderately bind to ThT (Fig. 5B) and CR dye (Fig. 5C). Furthermore, these isolated pure oligomers showed globular morphology (Fig. 5D).

**Fig 6:**
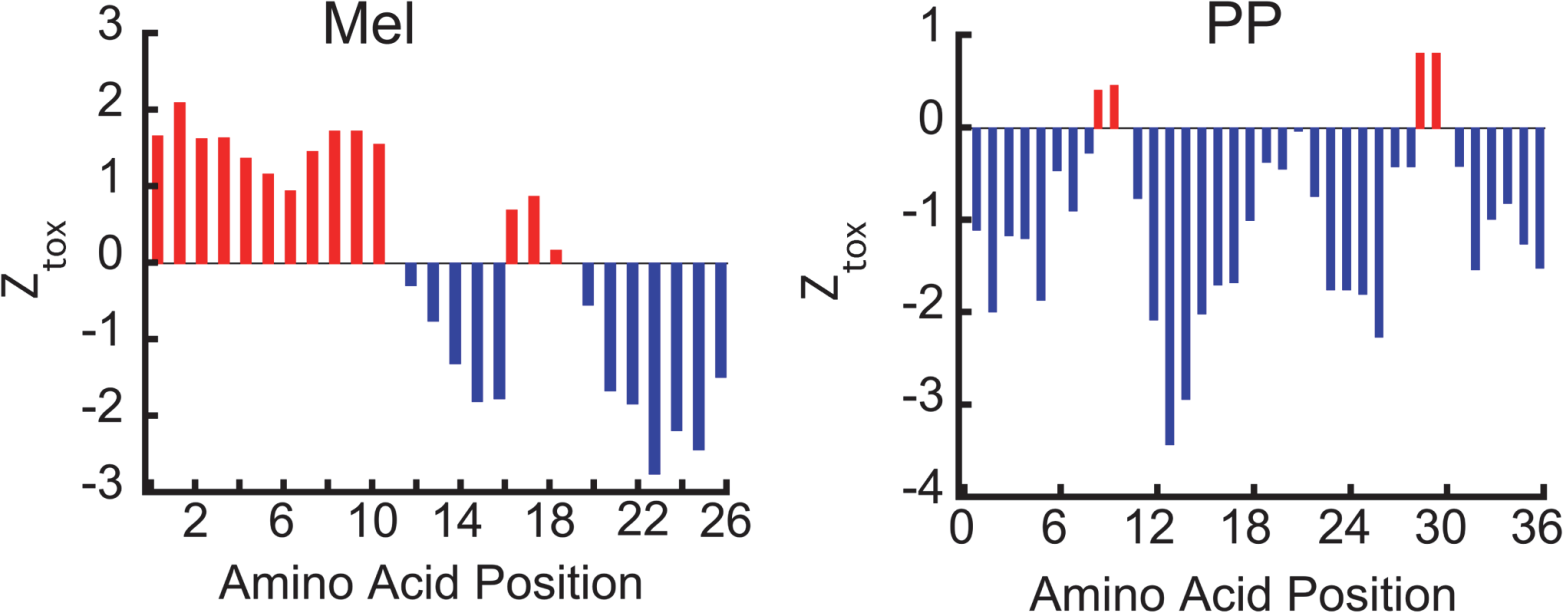
Oligomerization prediction of Mel and PP. The intrinsic oligomerization ability of Mel and PP peptide was calculated (at pH 5.5) using Zyggregator software. The positive values (in red) represent aggregation propensity of corresponding amino acid.

As Mel and PP oligomers (formed in the presence of heparin) showed tinctorial properties similar to amyloid, we, therefore, checked the intrinsic amyloidogenic propensity of these peptides using Zyggregator algorithm [37]. The Zyggregator prediction clearly showed that both Mel and PP possess intrinsic tendency to form amyloidogenic oligomers, however, this propensity is comparatively higher for Mel (Fig. 6). Although both PP and Mel formed oligomers in presence of heparin, which bind moderately to ThT and CR, at this point, it is not clear how helical oligomers, which lacked β-sheet structure bind to ThT and CR dye. It is possible that a fraction of both PP and Mel form amyloid fibrils (which may not detectable in CD and FTIR studies (Fig. 1), which may bind moderately to ThT and CR. For this, we examined the immunoreactivity of these oligomers with amyloid oligomer specific (A11) and amyloid fibril specific (OC) antibodies [5,38] using dot blot assay. Two weeks incubated peptide samples (in the presence and absence of heparin) were used for this study. The β-sheet rich AS oligomers and unstructured AS monomers (both isolated using SEC) were used as positive and negative controls, respectively. The A11 antibody showed immunoreactivity only with β-sheet rich AS oligomers (S4 Fig.). However, Mel and PP oligomers did not show any immunoreactivity with A11 antibody.

The data suggest that helical oligomers of PP and Mel might lack the epitopes for A11 antibody (S4 Fig.). When we examined the immunoreactivity of these samples with amyloid fibril specific OC-antibody [38] (S4 Fig.), which binds to β-sheet rich amyloid fibrils, the peptide oligomers were also found to be non-immunoreactive with OC antibody, suggesting the absence of β-sheet rich fibrillar aggregates in these samples. Unstructured AS monomers and preformed β-sheet rich AS fibrils were used as OC-negative and OC-positive controls, respectively. The peptides incubated in the absence of heparin were found negative for both A11 and OC immunoreactivity (S4 Fig.). The data suggest that irrespective of the absence of fibrils, these helix-rich oligomers may provide the structural milieu for binding with ThT and CR similar to amyloid fibrils. Previously, protein/peptide aggregates with helix-rich structure also showed both ThT and CR binding [46,47].

### The oligomers are cytotoxic to SH-SY5Y neuronal cells

**Fig 7:**
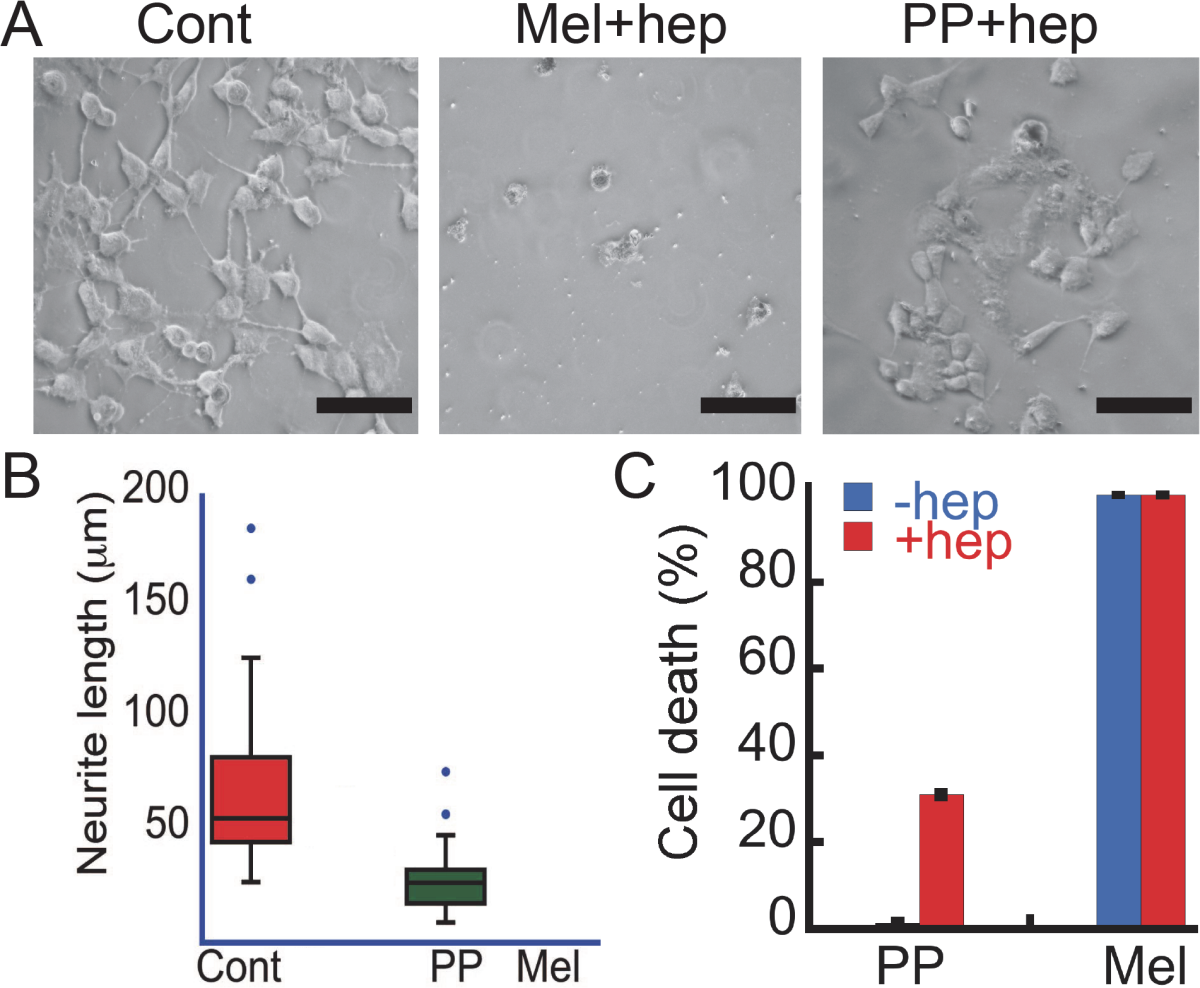
Cytotoxicity of Mel and PP oligomers. **(A)** Phase contrast images of SH-SY5Y cells showing damaged morphology of cells by PP and Mel oligomers. Scale bars are 100 μm. **(B)** Neurite length of SH-SY5Y cells treated with Mel and PP oligomers that were quantified using ImageJ software (NIH). The cells treated with oligomers showing reduced neurite length when compared to control. Due to complete death of cells in Mel oligomer treated samples, the calculation was only conducted for control samples (buffer) and PP oligomers treated samples. **(C)** LDH assay depicting % cell death in SH-SY5Y cells treated with oligomers. Both PP and Mel oligomers (formed in the presence of heparin) showed cytotoxicity. PP incubated in the absence of heparin did not show any toxicity; however, Mel incubated alone showed cytotoxicity.

Mel and PP oligomers (formed in the presence of heparin) showed tinctorial properties similar to PD associated AS oligomers. Therefore, the cytotoxicity of these oligomers was evaluated using SH-SY5Y cells. For cytotoxicity measurements, we performed morphological analysis of SH-SY5Y cells and LDH release assay in absence and presence of 10 μM oligomers. Our morphological analysis data revealed that after 30 h of treatment with PP oligomers, the number of cells was decreased compared to buffer control (Fig. 7A). Further analysis of cells showed significant loss of neuritic extensions as evident from the measurements of neuritic lengths in the presence of oligomers (measured using ImageJ software, NIH) (Fig. 7B). However, cells in the presence of Mel oligomers showed complete death and only cell debris were observed (Fig. 7A) and therefore we were unable to calculate neurite length of Mel treated cells. Further, concentration-dependent LDH assay was performed to quantify the cell death (Fig. 7C and S5 Fig.) induced by these oligomers. LDH is a soluble cytosolic enzyme that is released into the culture medium following the loss of membrane integrity and cell death [39]. This method is widely used to assay the toxicity of chemicals or environmental toxic factors on cells [39]. PP oligomers (10 μM), which were formed in the presence of heparin after two weeks of incubation showed ~ 35% cell death in LDH release assay (Fig. 7C). However, in similar conditions, PP incubated for two weeks in the absence of heparin did not show significant LDH release/cell death (Fig. 7C), suggesting that the toxicity of PP is a consequence of its oligomerization in presence of heparin.

Interestingly, our study revealed that 10 μM Mel oligomers (both formed in the presence and absence of heparin) after two weeks of incubation showed ~100% cytotoxicity in LDH assay, consistent with our cell morphology analysis. Previously, it has been shown that Mel has hemolytic activity and suggested that this activity is related to its oligomerization [48]. The morphological analysis and LDH data collectively suggest that the helical oligomers of Mel and PP are cytotoxic to SH-SY5Y cells. However, it is not clear at this point why the extent of toxicity by Mel oligomers formed in presence and absence of heparin is similar irrespective of their different oligomers sizes. The data suggest that the toxicity of Mel oligomers might not be correlated with their size prior to the addition into cell culture. We also compared the toxicity of freshly dissolved Mel (10 μM) (S6 Fig.) and unstructured Mel oligomers formed after two weeks of incubation in the absence of heparin (Fig. 7C). The LDH data showed that both preparations of Mel (freshly dissolved and two weeks incubated) were highly toxic (~100%) to SH-SY5Y similar to large oligomers formed in the presence of heparin. It is reported that cell surface glycosaminoglycans can induce structural transition of unstructured Mel into helix-rich conformation [25]. We believe that this toxicity may arise due to *in situ* helix-rich oligomer formation by Mel on the cell surface.

**Fig 8:**
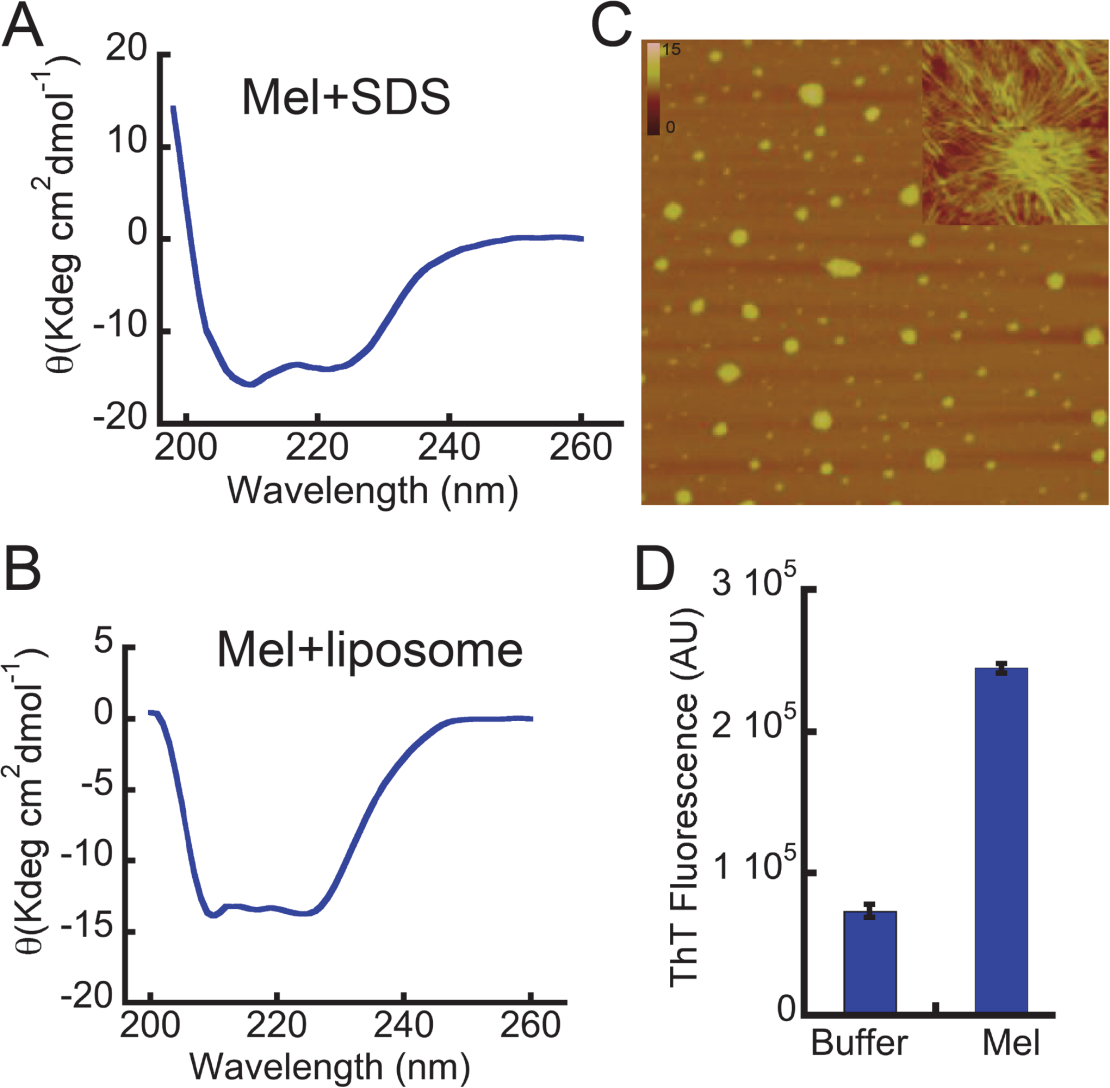
Biophysical characterization of Mel in presence of SDS and liposome. **(A)** CD spectroscopy showing the helical conformation of Mel after immediate addition of SDS (2.5 mM) in Gly-NaOH buffer (20 mM, pH 9.2). (**B)** Mel showing immediate conversion to helical conformation after addition of liposomes. **(C)** AFM images of Mel (incubated in the presence of SDS at 37°C) showing large globular oligomers and some fibrillar species (shown in the inset). **(D)** ThT binding of Mel after 5 days of incubation in the presence of SDS.

To study further that the cell membrane might have a role in structural change and oligomerization of Mel, we studied the Mel oligomerization in the presence of membrane-mimicking condition (SDS) and membrane vesicles (Fig. 8). Our data suggest that both of these conditions instantly promoted helix formation of Mel (Fig. 8A and 8B, respectively). To further analyze the oligomerization of Mel (25 μM) in the presence of SDS (2.5 mM), we performed morphological analysis of Mel after 5 days incubation in SDS. The AFM analysis showed the presence of fibrillar species along with the globular oligomers (Fig. 8C). Moreover, this aggregated Mel sample in the presence of SDS also showed tinctorial property like ThT fluorescence (Fig. 8D), suggesting that these aggregates are amyloidogenic in nature. It is interesting to note that ThT fluorescence was also observed on day 0 sample (S7 Fig.), suggesting that the addition of SDS may immediately induce the oligomerization of Mel. The data collectively suggests that like other amyloidogenic proteins, membrane-mimicking environment may also promote the self-assembly of Mel into oligomers.

### Hydrophobic surface exposure of oligomers

**Fig 9:**
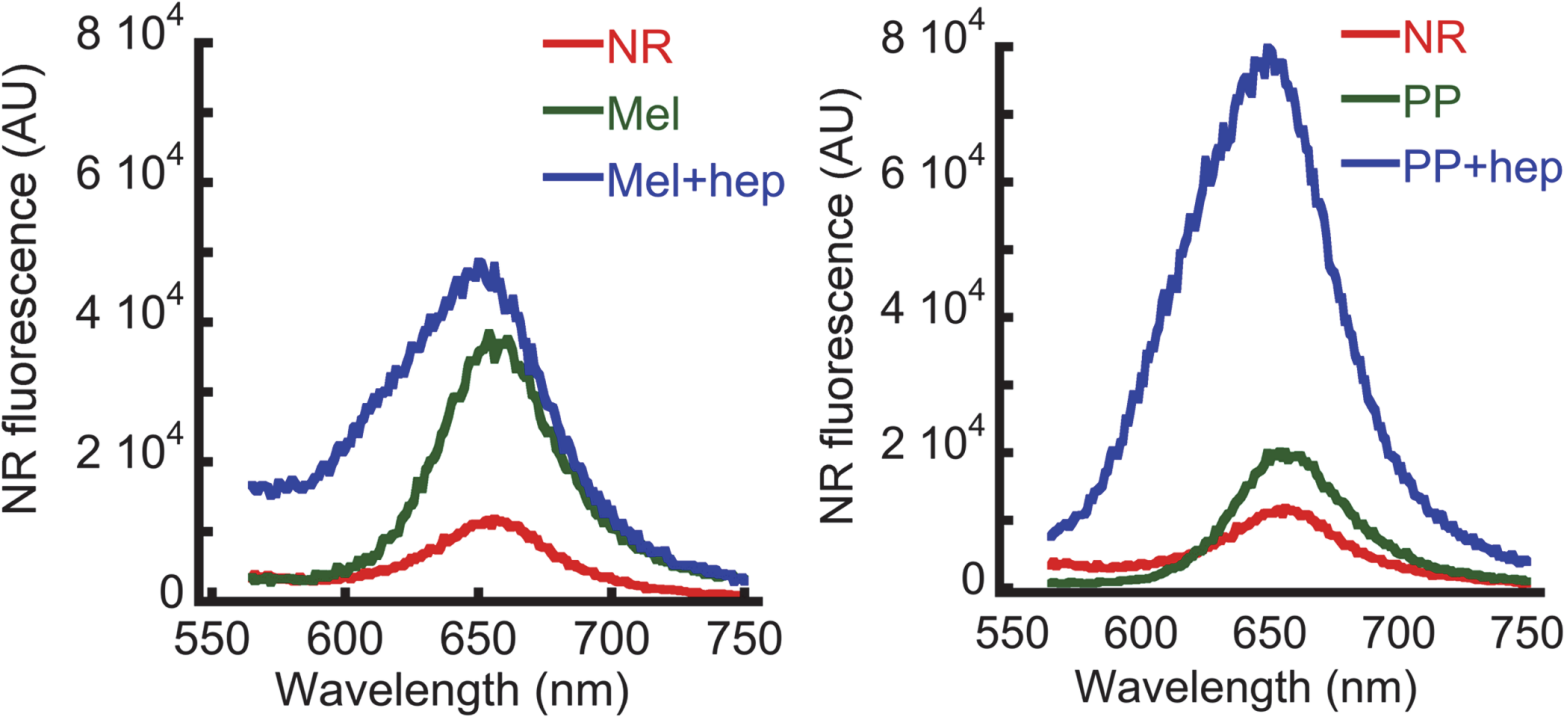
Hydrophobic surface exposure of oligomers. Hydrophobic surface exposure in terms of NR binding by Mel and PP samples, incubated for two weeks in presence and absence of heparin. The data suggesting increased hydrophobic surface exposure during heparin-induced peptide oligomerization.

It has been suggested that the extent of hydrophobic surface exposure may play a crucial role in cellular toxicity of protein aggregates [49,50]. We hypothesize that along with structural and morphological changes, oligomerization may induce hydrophobic surface exposure of the peptides that in turn promote their insertion in the cell membrane and thereby cytotoxicity. To test this, Nile Red (NR), which is a neutral dye and sensitive for detecting exposed hydrophobic surface of the protein/peptide [51,52] was used. The NR binding data showed a significantly high NR fluorescence intensity after binding to peptide oligomers formed in the presence of heparin compared to peptides incubated in the absence of heparin (Fig. 9). The present data thus suggest that due to oligomerization, the hydrophobic surface exposure of the protein/peptide increases, which then interacts and damages the cell membrane and eventually kills the cells.

### Peptide oligomers form ion channels and permeabilize the lipid vesicles

**Fig 10:**
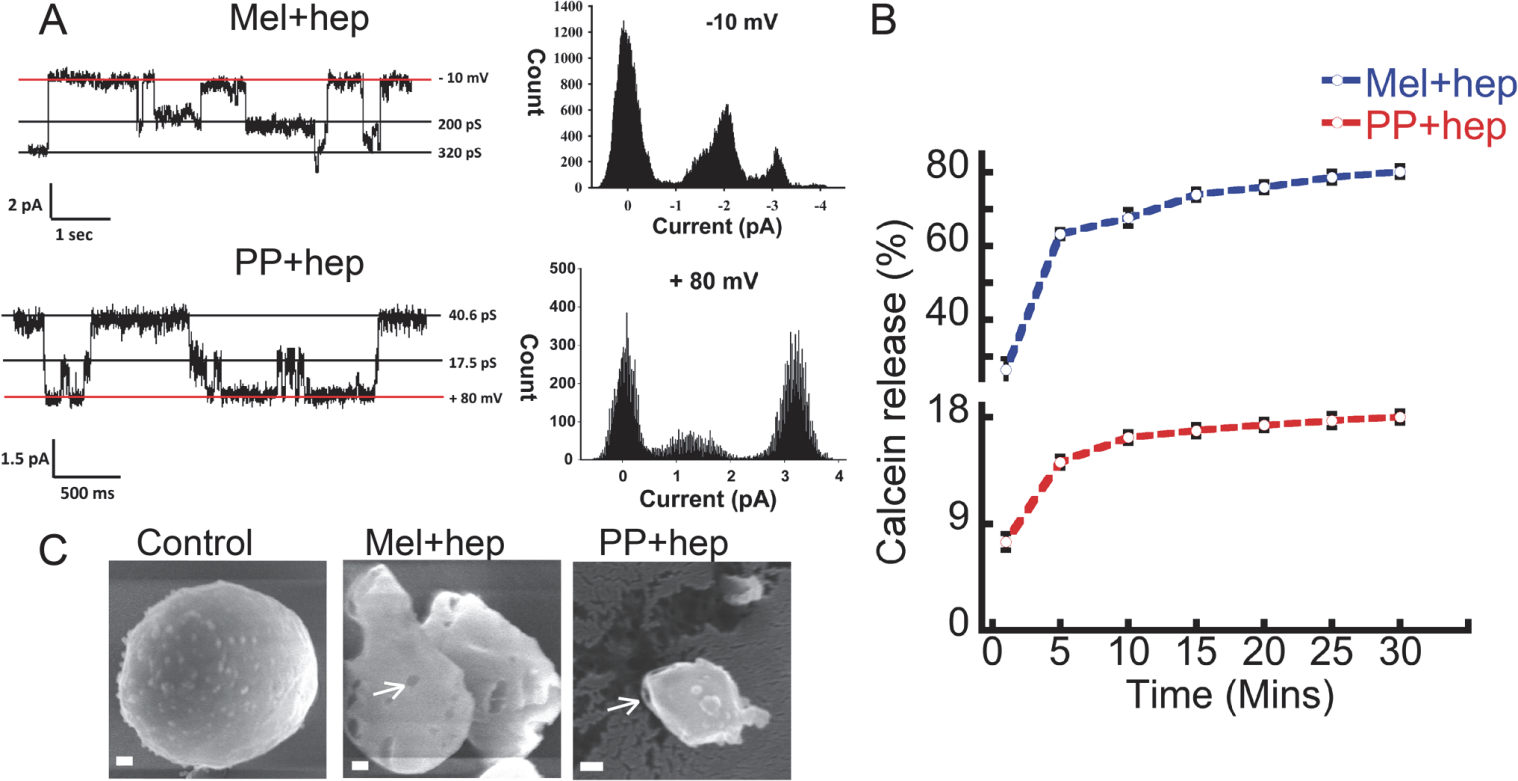
Membrane damage by oligomers. **(A)** Representative single channel current traces; exhibited by oligomeric species at different holding potentials. Channel insertion was initiated by adding 1 μM of Mel and PP oligomers to the cis chamber. All point histogram of the corresponding current trace is shown at the right side. Clamping potentials (mV) are indicated along the right side of the current traces. Conductance values (in pS) of different states are indicated on the right side of current traces. Red horizontal line represents a base line (0 pA). **(B)** Calcein release assay showing leakage of the calcein dye after addition of oligomers to the calcein-loaded liposomes. A high calcein fluorescence was observed when calcein was released to the solution. **(C)** Cryo-SEM images of liposomes showing the direct visualization of pore and membrane damage in the presence of oligomers. Arrows indicate the pore-like structures in the liposomes. Scale bars are 100 nm.

It was previously suggested that many amyloid oligomers permeabilize lipid bilayers and form ion channels in the cell membrane, thereby disruptimg the cellular homeostasis eventually causing amyloid diseases such as Alzheimer’s and Parkinson’s [53–55]. To further explore the toxicity mechanism of these oligomers, we analyzed whether Mel and PP oligomers, which were formed in the presence of heparin can form channel/pore in a model membrane. To do this, artificial bilayer was constructed from 1, 2-diphytamoyl-*sn*-glycero-3-phosphocholine (DPhPC). The peptide oligomers were added to these lipid membranes and the channel activity was monitored at different voltages. Our data showed that oligomers of PP and Mel (formed in presence of heparin) readily formed channels in artificial bilayer lipid membrane (BLM) within 5–10 min of their addition to cis chamber (Fig. 10A). In similar experimental condition, PP that was incubated in the absence of heparin, did not exhibit any channel activity (S8 Fig.), suggesting that channel formation activity of PP is associated with its oligomerization. In fully open state, single channel conductance of Mel oligomers (formed in the presence of heparin) was about 320 ± 0.04 pS (n = 7) in 1M KCl. Mel oligomers showed one prominent sub-conductance state of 200 ± 0.02 pS (n = 7). However, PP showed several sub-conductance states of which one of the 17.5 ± 0.04 pS (n = 3) was observed frequently. Mel oligomers, which were formed after two weeks of incubation in the absence of heparin also formed channel, however with approximately 30 times lesser single channel conductance, compared to Mel oligomers formed in presence of heparin (S8 Fig.).

To further reveal the channel forming capability of these oligomers, calcein release assay was performed using calcein-loaded liposomes. If these oligomers are forming channel/pores in the liposomes; the fluorescent calcein dye will leak out from the liposome in the solution. The leakage of calcein, from the liposomes, was detected by measuring the time-dependent calcein fluorescence in the solution (Fig. 10B). The addition of oligomers (10 μM) to the calcein-loaded liposomes at RT showed prominent increase of calcein fluorescence in the solution. For positive control (for 100% calcein release), 0.5% Triton X-100 was also used. The background calcein fluorescence from calcein-loaded liposomes was very less during the entire measurement time (30 min) and it was subtracted from the calcein fluorescence values obtained after oligomer treatment. Addition of Mel oligomers (formed in the presence of heparin) to calcein-loaded liposomes showed ~80% calcein release (as evident from calcein fluorescence in solution). Similarly, addition PP oligomers (formed in the presence of heparin) showed ~20% calcein fluorescence in solution (Fig. 10B). Two weeks incubated Mel alone sample induced ~ 60% calcein release, consistent with its toxic oligomer formation tendency. However, PP incubated for two weeks in the absence of heparin, showed negligible calcein fluorescence when added to calcein-loaded liposome solution (data not shown). The dye leakage assay, therefore, supports the electrical conductance data, suggesting channel/pore formation in the lipid vesicles by oligomers.

Furthermore, to visually observe any pore formation or membrane disruption by these different oligomers, we analyzed the morphology of liposomes in presence and absence of oligomers. The liposomes were incubated with 10 μM oligomers for 30 min at RT and the morphology of liposomes were analyzed using cryo-SEM (Fig. 10C). The data suggested that the oligomer treatment damaged/distorted the liposomes and some pores were also observed on the liposome (Fig. 10C). The electrical conductance, calcein release data, and liposome damage experiment collectively suggest that both Mel and PP oligomers interact with membrane (probably due to their exposed hydrophobic surfaces) and damage the membrane integrity, which subsequently lead to the leakage of inner content. Similar mechanism could be assumed for SH-SY5Y neuronal death in the presence of these oligomers.

### Comparison of Mel and PP oligomers with PD associated AS oligomers

**Fig 11:**
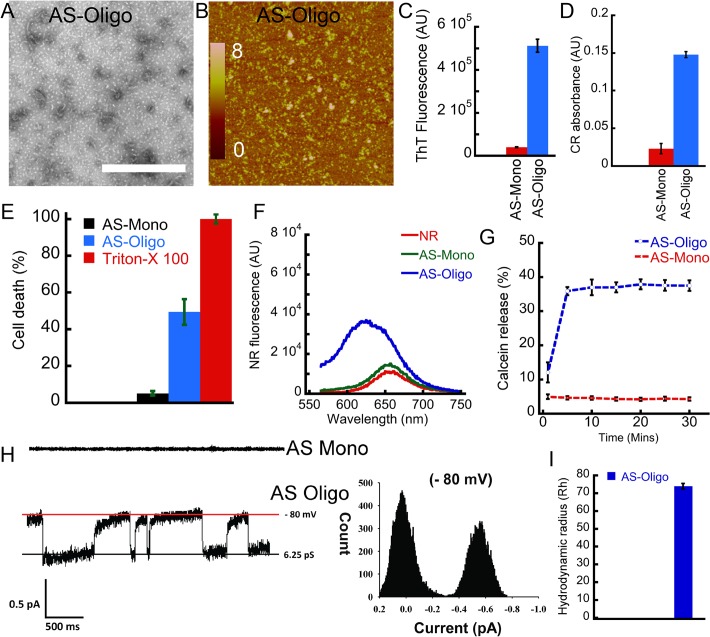
Biophysical characterization and cytotoxicity of AS oligomers. **(A)** EM **(B)** AFM images showing globular morphology with presence of some protofilaments by preformed AS oligomers (isolated from SEC). **(C)** ThT fluorescence and **(D)** CR absorbance of AS monomers and oligomers, respectively. **(E)** Toxicity assay measured by LDH showing ~ 45% cell death by AS oligomers; whereas no substantial toxicity was observed for AS monomers. **(F)** NR binding showing AS oligomers have higher NR binding (higher hydrophobic surface exposure) compared to the monomers. **(G)** Calcein release profile after addition of AS oligomers to calcein-loaded liposome solution. **(H)** Representative single channel current traces; exhibited by AS oligomers, suggesting channel formation in BLM. AS monomers did not show any channel activity. All point histogram of the corresponding current trace is presented at the right side. (**I)** Hydrodynamic radius (Rh) of AS oligomers (major population).

Mel and PP oligomers (formed in the presence of heparin) were cytotoxic and showed amyloid specific tinctorial properties (ThT and CR binding). Furthermore, Zyggregator calculation also suggested that these peptides possess an intrinsic amyloidogenic propensity. Therefore, we compared the biophysical properties of these oligomers with PD associated AS oligomers. We also compared the toxicity mechanism of Mel and PP oligomers with AS oligomers. For this purpose, preformed AS oligomers were isolated using SEC and used. Similar to Mel and PP oligomers, which were formed in presence of heparin, preformed AS oligomers also showed globular morphology along with some small protofilament like species under EM (Fig. 11A) and AFM (Fig. 11B). The high ThT (Fig. 11C) and CR binding (Fig. 11D) of these oligomers revealed their amyloidogenic nature. Furthermore, like Mel and PP oligomers, AS oligomers also induced death of SH-SY5Y cells in concentration-dependent manner. However, AS monomers did not show such toxic effect (Fig. 11E and S5 Fig.). Similarly, AS oligomers also have more exposed hydrophobic surfaces compared to monomeric AS (as measured by NR binding assay) (Fig. 11F). These data collectively suggest that the non-disease associated oligomers of Mel and PP share some biophysical parameters with PD associated AS oligomers.

Moreover, like PP and Mel oligomers, AS oligomers also released the calcein dye from calcein-loaded liposomes (Fig. 11G), suggesting a common mode of toxicity for these oligomers. Addition of AS oligomers to calcein-loaded liposome solution induced ~40% calcein release in solution (as evident from calcein fluorescence in solution). However, addition of AS monomers to calcein-loaded liposome solution showed a negligible increase in calcein fluorescence in solution (Fig. 11G). To further explore a common mode of toxicity, we also studied the channel activity of AS oligomers in BLM. The data suggests that AS oligomers also formed channels in BLM within 5–10 min of their addition to cis chamber in planar bilayer lipid recording (Fig. 11H), consistent with the previous observations of pore formation by amyloidogenic oligomers [54,55]. However, the channel conductance of AS oligomers was lesser than Mel and PP oligomers formed in the presence of heparin. In fully open state, single channel conductance of Mel was about 320 ± 0.04 pS (n = 7) in 1M KCl, whereas AS showed the conductance of about 6.25 ± 0.06 pS (n = 4). The monomeric AS did not show any channel activity in similar experimental condition (Fig. 11H). When we analyzed the Rh of oligomeric AS, we found that AS oligomeric sample has average Rh of 74±1.5 nm (Fig. 11I). Collectively, we found that not only the extent of toxicity but the other biophysical characteristics for toxicity mechanism were comparable for non-disease associated Mel, PP oligomers and PD associated AS oligomers.

## Discussion

The growing body of evidences suggests that the soluble protein/peptide oligomers are the most cytotoxic species causing cell death that occurs in neurodegenerative disorders including PD and AD [4–6,10,11,56,57]. Therefore, understanding the formation of these neurotoxic assemblies and determination of their structure-function relationship is important for the development of therapeutics against neurodegenerative diseases. Recent studies suggest that many unstructured peptides/proteins form short-lived helix-rich oligomers before converting into β-sheet rich fibrillar species [16,17]. These helical intermediates, which were initially observed in Aβ aggregation [16] are now shown to appear in the aggregation pathway of other amyloidogenic proteins like insulin, IAPP and other designed peptides [17]. It has been recently reported that IAPP (associated with type II diabetes) helical oligomers are able to promote significant apoptosis of pancreatic β cells [19]. Therefore, detailed biophysical characterization and understanding the mechanism of toxicity by helix-rich oligomers is important. However, due to their transient nature, the structure-toxicity study of helix-rich intermediate species is difficult to achieve. Therefore, peptides/proteins, which form amyloidogenic stable helical oligomers and possess cellular toxicity, could serve as a model system in this aspect.

In this work, we studied the structural transition and oligomerization of two different peptides (Mel and PP), both are known to possess stable helical fold [29,58,59]. Although Mel is known to possess an unstructured conformation, it is shown to transform into tetrameric helix-rich conformation in various conditions [27] and this transition is suggested to be responsible for its toxicity [48,59]. In contrast, PP is not known to possess any toxicity and/or oligomerization tendency. Moreover, the Zyggregator algorithm suggests that both PP and Mel possess oligomerization tendency (Fig. 6). Indeed our structural analysis (using CD and FTIR) (Fig. 1) and morphological analysis (by EM and AFM) (Fig. 2) suggest that both PP and Mel instantaneously oligomerized in presence of heparin. Heparin was used to induce the oligomerization of Mel and PP as this negatively charged glycosaminoglycan is well known to promote amyloid aggregation of many peptides/proteins irrespective of disease association [22,42,46,47,60–62].

Both Mel and PP also possess basic amino acid stretches, which may serve as a heparin-binding motif [41,42]. Further, it has been previously shown that heparin can induce the helix-rich conformation in Mel [25], suggesting that cell surface molecules such as heparin may play a significant role in its conformational transition and thereby toxicity. After two weeks of incubation, the size of Mel oligomers (in the presence of heparin) increased without any further conformational transition, suggesting that helical oligomers of Mel are stable (Figs. 1 and 2). Interestingly, when PP was incubated in the presence of heparin, it retained its helical conformation immediately after addition of heparin as well as after two weeks of incubation (Fig. 1), however, it assembled into globular oligomers in presence of heparin (Fig. 2). The data indicate that negatively charged heparin might increase the local concentration of both the peptides, which in turn promotes self-assembly through amphipathic helix-rich conformation.

Interestingly, in contrast to PP, Mel sample, which was incubated in the absence of heparin also showed some oligomeric assemblies (S3 Fig.). However, this oligomerization was not accompanied by any structural transition because two weeks incubated Mel remained mostly unstructured (Fig. 1). The data suggest that Mel is intrinsically more oligomerization prone compared to PP, consistent with our oligomerization prediction, where we found that many residues of N-terminus of Mel has aggregation propensity (Fig. 6). It is remarkable to note that both Mel and PP oligomers retained their helical conformations even after long incubation, indicating that stable helical conformations of these oligomers preclude their further conformation transition into β-sheet rich fibrillar aggregates.

The toxicity data suggest that both Mel and PP oligomers formed in the presence of heparin are highly cytotoxic (Fig. 7 and S5 Fig.). Interestingly, unstructured Mel oligomers formed in the absence of heparin and unstructured monomeric Mel also showed toxicity, similar to helical oligomers of Mel formed in the presence of heparin (Fig. 7). We propose that Mel is capable of oligomerizing instantaneously and can form helix-rich oligomers either in the presence of cell surface glycosaminoglycans or in the vicinity of the cell membrane. Consistent with this, it has been previously shown that Mel transforms its unstructured conformation into helical conformation in the presence of heparin [25]. Our structural studies of Mel using CD spectroscopy, in presence of membrane mimicking condition and in the presence of membrane vesicle, suggested that Mel transformed into helical conformation immediately in these conditions and also showed mostly globular oligomers (Fig. 8) and thus support our hypothesis.

Both PP and Mel oligomers lack any β-sheet rich structure; however, they possess tinctorial properties and cytotoxicity similar to PD associated AS oligomers. Furthermore, similar to AS oligomers, Mel and PP oligomers showed exposed hydrophobic surfaces. Therefore, we suggest that exposed hydrophobic surfaces of these oligomers might enable them to interact with the cell membrane and initiate cell death by altering the membrane integrity. It has been shown previously that many amyloid oligomers interact with membrane and initiate channel/pore formation, which subsequently leads to disruption of membrane integrity, leakage of cellular content and thereby cell death [53–55]. Mel oligomers, which formed in the absence of heparin, showed lesser single channel conductance compared to Mel oligomers formed in the presence of heparin. However, both kinds of Mel oligomers (formed in the presence and absence of heparin) showed ~100% cell death in LDH assay. This discrepancy might result due to the difference in the experimental parameters used in both the experiments. In BLM channel activity measurement, the oligomer treatment was done for 30 min, whereas, in LDH toxicity assay, the SH-SY5Y cells were exposed to oligomers for 30 h before quantifying the cell death. Therefore, this sufficiently longer exposure of Mel oligomers (formed in the presence and absence of heparin) to SH-SY5Y cells (30 h) resulted in 100% cell death, despite differences in their single channel conductance. Furthermore, as cell death was quantified by measuring the release of cellular LDH in the solution, it could be possible that despite differences in the channel size, the extended treatment time allowed equal amount of cellular LDH release for both kinds of oligomers.

Many recent studies suggest that intermediate oligomeric species possess higher cellular toxicity compared to mature amyloid fibrils [63]. For example, recently we have also shown that the preformed AS oligomers (isolated from SEC), which we used in this study, induced more neuronal death compared to a similar concentration of AS fibrils in cell culture [35]. Our concentration-dependent toxicity assay also revealed that Mel oligomers have higher toxicity compared to AS oligomers, whereas PP oligomers possess lesser toxicity compared to AS oligomers (S5 Fig.). The data collectively revealed that amyloidogenic oligomers, irrespective of their disease association, exert cell death by forming membrane channels/pores.

## Conclusion

The present study showed the formation of stable helix-rich cytotoxic globular oligomers of Mel and PP in presence of heparin. These oligomers showed amyloid-specific tinctorial properties, however, they did not further convert into β-sheet rich fibrils. We also found that similar to PD associated AS oligomers, Mel and PP oligomers possess hydrophobic surface exposure and membrane channel formation ability. Since these oligomers are stable in nature, they could be used as model systems for detailed biophysical characterization and high-resolution structural analysis of helical oligomers.

## Supporting Information

S1 Fig Amino acid sequence of pancreatic polypeptide (PP) and melittin (Mel). (TIF)

S2 Fig AFM analysis of Mel and PP samples (in the absence and presence of heparin) on day 0. In the absence of heparin, Mel and PP did not show oligomers, however, showed a considerable amount of oligomeric population after addition of heparin on day 0.
(TIF)

S3 Fig Morphological characterization of Mel and PP incubated in the absence of heparin for two weeks. In the absence of heparin, Mel and PP did not show oligomers, however, showed a considerable amount of oligomeric population after addition of heparin on day 0.
(TIF)

S4 Fig Dot blot assay of two Mel and PP oligomers. Mel and PP samples incubated for two weeks (in absence and presence of heparin) using oligomer specific A11 antibody and fibril specific OC antibody. Mel and PP oligomers did not show any immunoreactivity with either A11 or OC antibody. AS monomers, oligomers and fibrils were used as controls.
(TIF)

S5 Fig Dose-dependent oligomer toxicity in SH-SY5Y cells. Different concentrations of oligomers (2.5 μM, 5.0 μM and 10 μM) were exposed to SH-SY5Y cells in cell culture for 30 h and then LDH assay was performed to quantify the cell death. Different concentrations of AS monomers were used as control.
(TIF)

S6 Fig Cytotoxicity measurement of freshly dissolved Mel. Cytotoxicity of freshly dissolved Mel (10 μM) was measured using LDH assay in SH-SY5Y cells. Triton-X-100 (0.5%) was used as positive control.
(TIF)

S7 Fig ThT fluorescence of Mel in the presence of SDS. ThT fluorescence of Mel (day 0) after addition of SDS. SDS (2.5 mM) was added to Mel solution (25 μM) and then ThT fluorescence spectrum was recorded immediately after addition of ThT to this sample (d0).
(TIF)

S8 Fig Representative single channel current traces recorded for Mel and PP samples (in the absence of heparin). In the recording of single channel current, PP sample (incubated for two weeks in the absence of heparin) did not show any channel activity. However, Mel (incubated for two weeks in the absence of heparin) showed channel activity.
(TIF)

## References

[1] Chiti F, Dobson CM. Protein misfolding, functional amyloid, and human disease. Annu. Rev. Biochem. 2006; 75: 333–66. 16756495 10.1146/annurev.biochem.75.101304.123901

[2] Maji SK, Wang L, Greenwald J, Riek R. Structure-activity relationship of amyloid fibrils. FEBS Lett. 2009; 583: 2610–7. 10.1016/j.febslet.2009.07.003 19596006

[3] Hardy J, Selkoe DJ. The amyloid hypothesis of Alzheimer's disease: progress and problems on the road to therapeutics. Science. 2002; 297: 353–6. 12130773 10.1126/science.1072994

[4] Winner B, Jappelli R, Maji SK, Desplats PA, Boyer L, Aigner S, et al In vivo demonstration that α-synuclein oligomers are toxic. Proc. Natl. Acad. Sci. U. S. A. 2011; 108: 4194–9. 10.1073/pnas.1100976108 21325059 PMC3053976

[5] Kayed R, Head E, Thompson JL, McIntire TM, Milton SC, Cotman CW, et al Common structure of soluble amyloid oligomers implies common mechanism of pathogenesis. Science. 2003; 300: 486–9. 12702875 10.1126/science.1079469

[6] Karpinar DP, Balija MB, Kugler S, Opazo F, Rezaei-Ghaleh N, Wender N, et al Pre-fibrillar α-synuclein variants with impaired β-structure increase neurotoxicity in Parkinson's disease models. EMBO J. 2009; 28: 3256–68. 10.1038/emboj.2009.257 19745811 PMC2771093

[7] De Felice FG, Vieira MN, Saraiva LM, Figueroa-Villar JD, Garcia-Abreu J, Liu R, et al Targeting the neurotoxic species in Alzheimer's disease: inhibitors of Aβ oligomerization. FASEB J. 2004; 18: 1366–72. 15333579 10.1096/fj.04-1764com

[8] Harper JD, Wong SS, Lieber CM, Lansbury PT. Observation of metastable Aβ amyloid protofibrils by atomic force microscopy. Chem. Biol. 1997; 4: 119–25. 9190286 10.1016/s1074-5521(97)90255-6

[9] Walsh DM, Lomakin A, Benedek GB, Condron MM, Teplow DB. Amyloid β-protein fibrillogenesis—Detection of a protofibrillar intermediate. J. Biol. Chem. 1997; 272: 22364–72. 9268388 10.1074/jbc.272.35.22364

[10] Kirkitadze MD, Bitan G, Teplow DB. Paradigm shifts in Alzheimer's disease and other neurodegenerative disorders: The emerging role of oligomeric assemblies. J. Neurosci. Res. 2002; 69: 567–77. 12210822 10.1002/jnr.10328

[11] Walsh DM, Klyubin I, Fadeeva JV, Cullen WK, Anwyl R, Wolfe MS, et al Naturally secreted oligomers of amyloid β protein potently inhibit hippocampal long-term potentiation *in vivo* . Nature. 2002; 416: 535–9. 11932745 10.1038/416535a

[12] Bitan G, Kirkitadze MD, Lomakin A, Vollers SS, Benedek GB, Teplow DB. Amyloid β-protein (Aβ) assembly: Aβ40 and Aβ42 oligomerize through distinct pathways. Proc. Natl. Acad. Sci. U. S. A. 2003; 100: 330–5. 12506200 10.1073/pnas.222681699 PMC140968

[13] Vieira MN, Forny-Germano L, Saraiva LM, Sebollela A, Martinez AM, Houzel JC, et al Soluble oligomers from a non-disease related protein mimic Aβ-induced tau hyperphosphorylation and neurodegeneration. J. Neurochem. 2007; 103: 736–48. 17727639 10.1111/j.1471-4159.2007.04809.x

[14] Bucciantini M, Giannoni E, Chiti F, Baroni F, Formigli L, Zurdo J, et al Inherent toxicity of aggregates implies a common mechanism for protein misfolding diseases. Nature. 2002; 416: 507–11. 11932737 10.1038/416507a

[15] Uversky VN, Fink AL. Conformational constraints for amyloid fibrillation: the importance of being unfolded. Biochim. Biophys. Acta. 2004; 1698: 131–53. 15134647 10.1016/j.bbapap.2003.12.008

[16] Kirkitadze MD, Condron MM, Teplow DB. Identification and characterization of key kinetic intermediates in amyloid β-protein fibrillogenesis. J. Mol. Biol. 2001; 312: 1103–19. 11580253 10.1006/jmbi.2001.4970

[17] Abedini A, Raleigh DP. A critical assessment of the role of helical intermediates in amyloid formation by natively unfolded proteins and polypeptides. Protein Eng. Des. Sel. 2009; 22: 453–9. 10.1093/protein/gzp036 19596696 PMC2719502

[18] Abedini A, Raleigh DP. A role for helical intermediates in amyloid formation by natively unfolded polypeptides? Phys. Biol. 2009; 6: 015005 10.1088/1478-3975/6/1/015005 19208933 PMC3215505

[19] Bram Y, Frydman-Marom A, Yanai I, Gilead S, Shaltiel-Karyo R, Amdursky N, et al Apoptosis induced by islet amyloid polypeptide soluble oligomers is neutralized by diabetes-associated specific antibodies. Sci. Rep. 2014; 4: 4267 10.1038/srep04267 24589570 PMC3940978

[20] Anderson VL, Ramlall TF, Rospigliosi CC, Webb WW, Eliezer D. Identification of a helical intermediate in trifluoroethanol-induced α-synuclein aggregation. Proc. Natl. Acad. Sci. U. S. A. 2010; 107: 18850–5. 10.1073/pnas.1012336107 20947801 PMC2973859

[21] Volles MJ, Lansbury PT Jr. Zeroing in on the pathogenic form of α-synuclein and its mechanism of neurotoxicity in Parkinson's disease. Biochemistry. 2003; 42: 7871–8. 12834338 10.1021/bi030086j

[22] Maji SK, Perrin MH, Sawaya MR, Jessberger S, Vadodaria K, Rissman RA, et al Functional amyloids as natural storage of peptide hormones in pituitary secretory granules. Science. 2009; 325: 328–32. 10.1126/science.1173155 19541956 PMC2865899

[23] Cavari S, Vannucchi S. Glycosaminoglycans exposed on the endothelial cell surface. Binding of heparin-like molecules derived from serum. FEBS Lett. 1993; 323: 155–8. 8495730 10.1016/0014-5793(93)81469-g

[24] Naik RJ, Chatterjee A, Ganguli M. Different roles of cell surface and exogenous glycosaminoglycans in controlling gene delivery by arginine-rich peptides with varied distribution of arginines. Biochim. Biophys. Acta. 2013; 1828: 1484–93. 10.1016/j.bbamem.2013.02.010 23454086

[25] Klocek G, Seelig J. Melittin interaction with sulfated cell surface sugars. Biochemistry. 2008; 47: 2841–9. 10.1021/bi702258z 18220363

[26] Habermann E. Bee and wasp venoms. Science. 1972; 177: 314–22. 4113805 10.1126/science.177.4046.314

[27] Wilcox W, Eisenberg D. Thermodynamics of melittin tetramerization determined by circular dichroism and implications for protein folding. Protein Sci. 1992; 1: 641–53. 1304363 10.1002/pro.5560010510 PMC2142234

[28] Batterham RL, Le Roux CW, Cohen MA, Park AJ, Ellis SM, Patterson M, et al Pancreatic polypeptide reduces appetite and food intake in humans. J. Clin. Endocrinol. Metab. 2003; 88: 3989–92. 12915697 10.1210/jc.2003-030630

[29] Gehlert DR. Multiple receptors for the pancreatic polypeptide (PP-fold) family: physiological implications. Proc. Soci. Exp. Biol. Med. 1998; 218: 7–22. 9572148 10.3181/00379727-218-44263

[30] Bouchard M, Zurdo J, Nettleton EJ, Dobson CM, Robinson CV. Formation of insulin amyloid fibrils followed by FTIR simultaneously with CD and electron microscopy. Protein Sci. 2000; 9: 1960–7. 11106169 10.1110/ps.9.10.1960 PMC2144465

[31] De Jong KL, Incledon B, Yip CM, DeFelippis MR. Amyloid fibrils of glucagon characterized by high-resolution atomic force microscopy. Biophys. J. 2006; 91: 1905–14. 16766610 10.1529/biophysj.105.077438 PMC1544305

[32] Ghosh D, Sahay S, Ranjan P, Salot S, Mohite GM, Singh PK, et al The newly discovered Parkinson's Disease associated Finnish mutation (A53E) attenuates α-synuclein aggregation and membrane binding. Biochemistry. 2014; 53: 6419–21 10.1021/bi5010365 25268550

[33] Volles MJ, Lansbury PT Jr. Relationships between the sequence of α-synuclein and its membrane affinity, fibrillization propensity, and yeast toxicity. J. Mol. Biol. 2007; 366: 1510–22. 17222866 10.1016/j.jmb.2006.12.044 PMC1868670

[34] Ghosh D, Mondal M, Mohite GM, Singh PK, Ranjan P, Anoop A, et al The Parkinson's disease-associated H50Q mutation accelerates α-synuclein aggregation in vitro. Biochemistry. 2013; 52: 6925–7. 10.1021/bi400999d 24047453

[35] Singh PK, Kotia V, Ghosh D, Mohite GM, Kumar A, Maji SK. Curcumin modulates α-synuclein aggregation and toxicity. ACS Chem. Neurosci. 2013; 4: 393–407. 10.1021/cn3001203 23509976 PMC3605819

[36] Maji SK, Schubert D, Rivier C, Lee S, Rivier JE, Riek R. Amyloid as a depot for the formulation of long-acting drugs. PLoS Biol. 2008; 6: e17 10.1371/journal.pbio.0060017 18254658 PMC2225439

[37] Tartaglia GG, Vendruscolo M. The Zyggregator method for predicting protein aggregation propensities. Chem. Soc. Rev. 2008; 37: 1395–401. 10.1039/b706784b 18568165

[38] Kayed R, Head E, Sarsoza F, Saing T, Cotman CW, Necula M, et al Fibril specific, conformation dependent antibodies recognize a generic epitope common to amyloid fibrils and fibrillar oligomers that is absent in prefibrillar oligomers. Mol. Neurodegener. 2007; 2: 18 17897471 10.1186/1750-1326-2-18 PMC2100048

[39] Behl C, Davis JB, Lesley R, Schubert D. Hydrogen peroxide mediates amyloid β protein toxicity. Cell. 1994; 77: 817–27. 8004671 10.1016/0092-8674(94)90131-7

[40] Krishnan R, Goodman JL, Mukhopadhyay S, Pacheco CD, Lemke EA, Deniz AA, et al Conserved features of intermediates in amyloid assembly determine their benign or toxic states. Proc. Natl. Acad. Sci. U. S. A. 2012; 109: 11172–7. 10.1073/pnas.1209527109 22745165 PMC3396487

[41] Hileman RE, Fromm JR, Weiler JM, Linhardt RJ. Glycosaminoglycan-protein interactions: definition of consensus sites in glycosaminoglycan binding proteins. Bioessays. 1998; 20: 156–67. 9631661 10.1002/(SICI)1521-1878(199802)20:2<156::AID-BIES8>3.0.CO;2-R

[42] Jha NN, Anoop A, Ranganathan S, Mohite GM, Padinhateeri R, Maji SK. Characterization of amyloid formation by glucagon-like peptides: role of basic residues in heparin-mediated aggregation. Biochemistry. 2013; 52: 8800–10. 10.1021/bi401398k 24236650

[43] LeVine H 3rd. Quantification of β-sheet amyloid fibril structures with thioflavin T. Methods Enzymol. 1999; 309: 274–84. 10507030 10.1016/s0076-6879(99)09020-5

[44] Klunk WE, Jacob RF, Mason RP. Quantifying amyloid by congo red spectral shift assay. Methods Enzymol. 1999; 309: 285–305. 10507031 10.1016/s0076-6879(99)09021-7

[45] Ghosh D, Dutta P, Chakraborty C, Singh PK, Anoop A, Jha NN, et al Complexation of amyloid fibrils with charged conjugated polymers. Langmuir. 2014; 30: 3775–86. 10.1021/la404739f 24678792

[46] Anoop A, Ranganathan S, Das Dhaked B, Jha NN, Pratihar S, Ghosh S, et al Elucidating the role of disulfide bond on amyloid formation and fibril reversibility of somatostatin-14: relevance to its storage and secretion. J. Biol. Chem. 2014; 289: 16884–903. 10.1074/jbc.M114.548354 24782311 PMC4059132

[47] Singh PK, Maji SK. Amyloid-like fibril formation by tachykinin neuropeptides and its relevance to amyloid β-protein aggregation and toxicity. Cell Biochem. Biophys. 2012; 64: 29–44. 10.1007/s12013-012-9364-z 22628076

[48] Klocek G, Schulthess T, Shai Y, Seelig J. Thermodynamics of melittin binding to lipid bilayers. Aggregation and pore formation. Biochemistry. 2009; 48: 2586–96. 10.1021/bi802127h 19173655

[49] Bolognesi B, Kumita JR, Barros TP, Esbjorner EK, Luheshi LM, Crowther DC, et al ANS binding reveals common features of cytotoxic amyloid species. ACS Chem. Biol. 2010; 5: 735–40. 10.1021/cb1001203 20550130

[50] Campioni S, Mannini B, Zampagni M, Pensalfini A, Parrini C, Evangelisti E, et al A causative link between the structure of aberrant protein oligomers and their toxicity. Nat. Chem. Biol. 2010; 6: 140–7. 10.1038/nchembio.283 20081829

[51] Sackett DL, Wolff J. Nile red as a polarity-sensitive fluorescent probe of hydrophobic protein surfaces. Anal. Biochem. 1987; 167: 228–34. 3442318 10.1016/0003-2697(87)90157-6

[52] Daban JR, Samso M, Bartolome S. Use of nile red as a fluorescent probe for the study of the hydrophobic properties of protein-sodium dodecyl sulfate complexes in solution. Anal. Biochem. 1991; 199: 162–8. 1812781 10.1016/0003-2697(91)90084-7

[53] Prangkio P, Yusko EC, Sept D, Yang J, Mayer M. Multivariate analyses of amyloid-β oligomer populations indicate a connection between pore formation and cytotoxicity. PLoS One. 2012; 7: e47261 10.1371/journal.pone.0047261 23077580 PMC3471831

[54] Kayed R, Sokolov Y, Edmonds B, McIntire TM, Milton SC, Hall JE, et al Permeabilization of lipid bilayers is a common conformation-dependent activity of soluble amyloid oligomers in protein misfolding diseases. J. Biol. Chem. 2004; 279: 46363–6. 15385542 10.1074/jbc.C400260200

[55] Quist A, Doudevski I, Lin H, Azimova R, Ng D, Frangione B, et al Amyloid ion channels: a common structural link for protein-misfolding disease. Proc. Natl. Acad. Sci. U. S. A. 2005; 102: 10427–32. 16020533 10.1073/pnas.0502066102 PMC1180768

[56] Lashuel HA, Lansbury PT Jr. Are amyloid diseases caused by protein aggregates that mimic bacterial pore-forming toxins? Q. Rev. Biophys. 2006; 39: 167–201. 16978447 10.1017/S0033583506004422

[57] Goldberg MS, Lansbury PT. Is there a cause-and-effect relationship between α-synuclein fibrillization and Parkinson's disease? Nature Cell Biol. 2000; 2: E115–E9. 10878819 10.1038/35017124

[58] Ladokhin AS, White SH. Folding of amphipathic α-helices on membranes: energetics of helix formation by melittin. J. Mol. Biol. 1999; 285: 1363–9. 9917380 10.1006/jmbi.1998.2346

[59] Terwilliger TC, Weissman L, Eisenberg D. The structure of melittin in the form I crystals and its implication for melittin's lytic and surface activities. Biophys. J. 1982; 37: 353–61. 7055627 10.1016/S0006-3495(82)84683-3 PMC1329151

[60] Suk JY, Zhang F, Balch WE, Linhardt RJ, Kelly JW. Heparin accelerates gelsolin amyloidogenesis. Biochemistry. 2006; 45: 2234–42. 16475811 10.1021/bi0519295 PMC2657342

[61] Goedert M, Jakes R, Spillantini MG, Hasegawa M, Smith MJ, Crowther RA. Assembly of microtubule-associated protein tau into Alzheimer-like filaments induced by sulphated glycosaminoglycans. Nature. 1996; 383: 550–3. 8849730 10.1038/383550a0

[62] Ranganathan S, Singh PK, Singh U, Singru PS, Padinhateeri R, Maji SK. Molecular interpretation of ACTH-β-endorphin coaggregation: relevance to secretory granule biogenesis. PLoS One. 2012; 7: e31924 10.1371/journal.pone.0031924 22403619 PMC3293876

[63] Chimon S, Shaibat MA, Jones CR, Calero DC, Aizezi B, Ishii Y. Evidence of fibril-like β-sheet structures in a neurotoxic amyloid intermediate of Alzheimer's β-amyloid. Nat. Struct. Mol. Biol. 2007; 14: 1157–64. 18059284 10.1038/nsmb1345

