# Supplementary figures and images for: Cytotoxic Helix-Rich Oligomer Formation by Melittin and Pancreatic Polypeptide
Source: PLoS One. 2015 Mar 24;10(3):e0120346. doi: 10.1371/journal.pone.0120346 (PMC4372375; doi:10.1371/journal.pone.0120346)

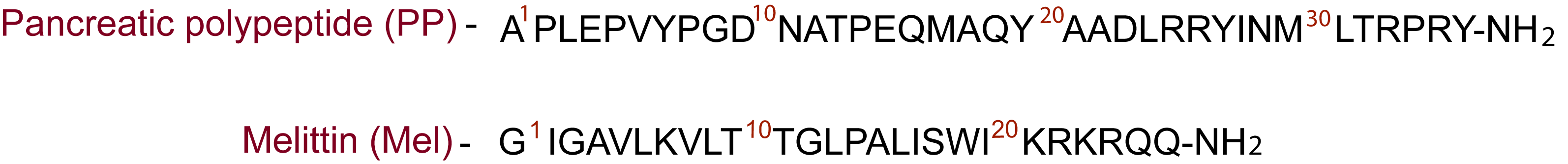

Supplement: S1 Fig — (TIF) [file pone.0120346.s001.tif]

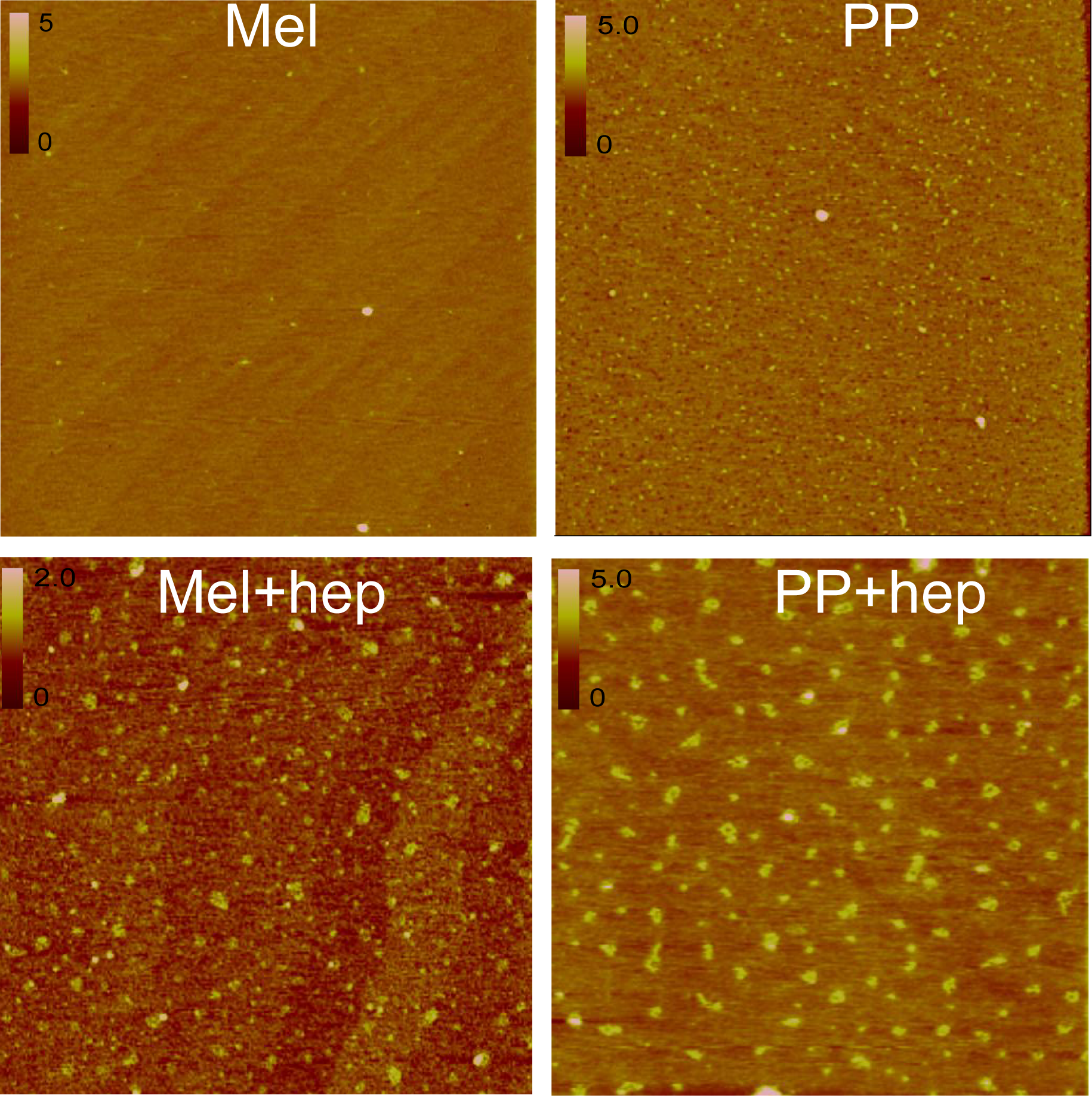

Supplement: S2 Fig — In the absence of heparin, Mel and PP did not show oligomers, however, showed a considerable amount of oligomeric population after addition of heparin on day 0. (TIF) [file pone.0120346.s002.tif]

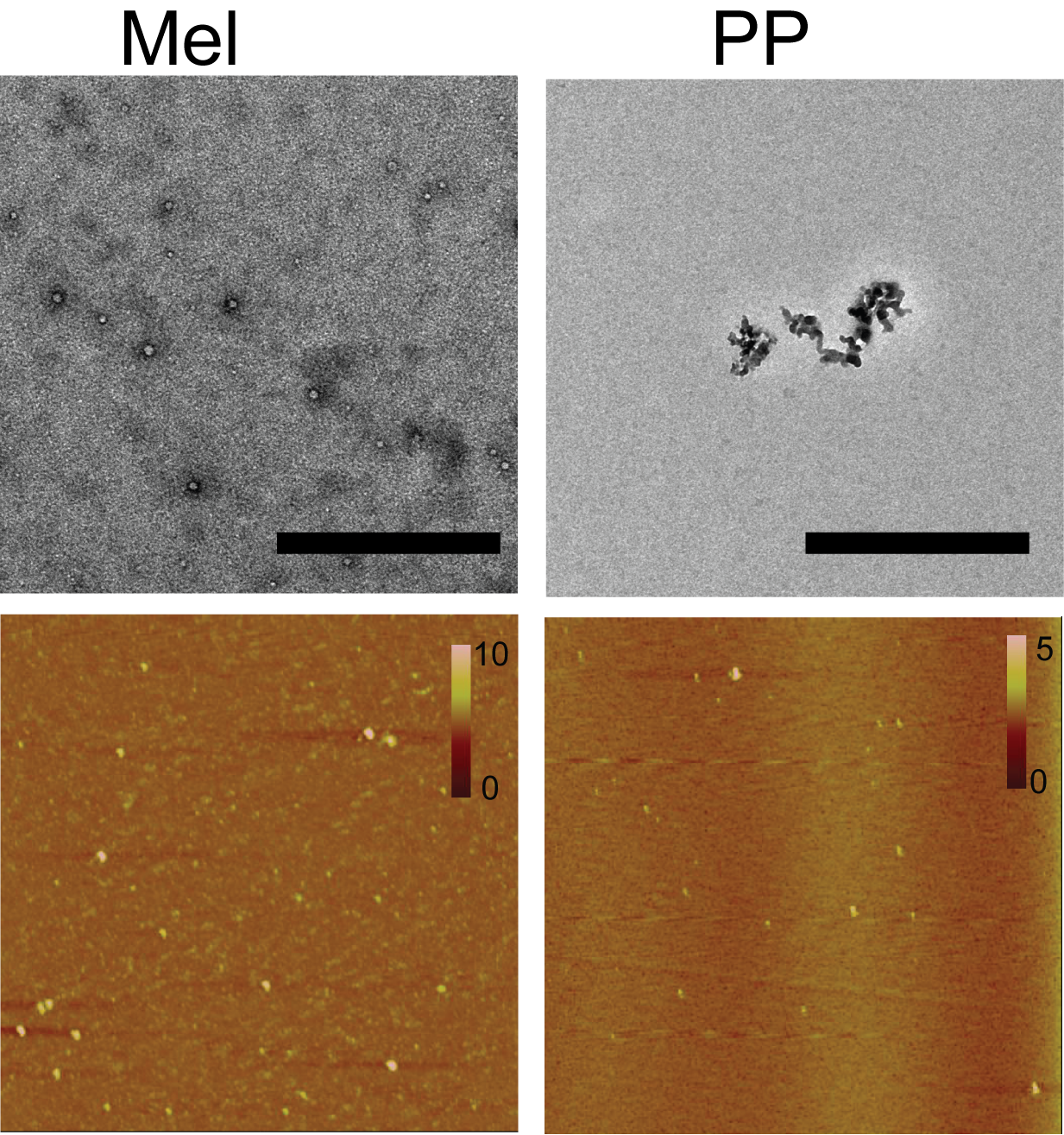

Supplement: S3 Fig — In the absence of heparin, Mel and PP did not show oligomers, however, showed a considerable amount of oligomeric population after addition of heparin on day 0. (TIF) [file pone.0120346.s003.tif]

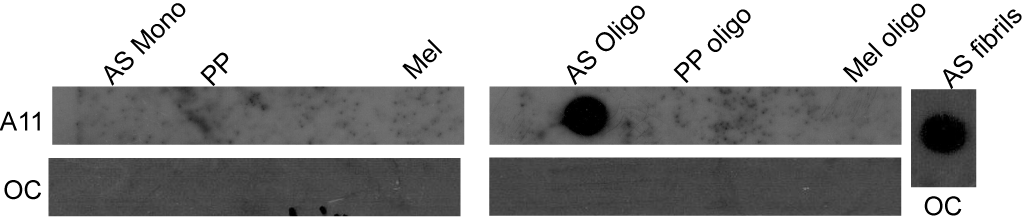

Supplement: S4 Fig — Mel and PP samples incubated for two weeks (in absence and presence of heparin) using oligomer specific A11 antibody and fibril specific OC antibody. Mel and PP oligomers did not show any immunoreactivity with either A11 or OC antibody. AS monomers, oligomers and fibrils were used as controls. (TIF) [file pone.0120346.s004.tif]

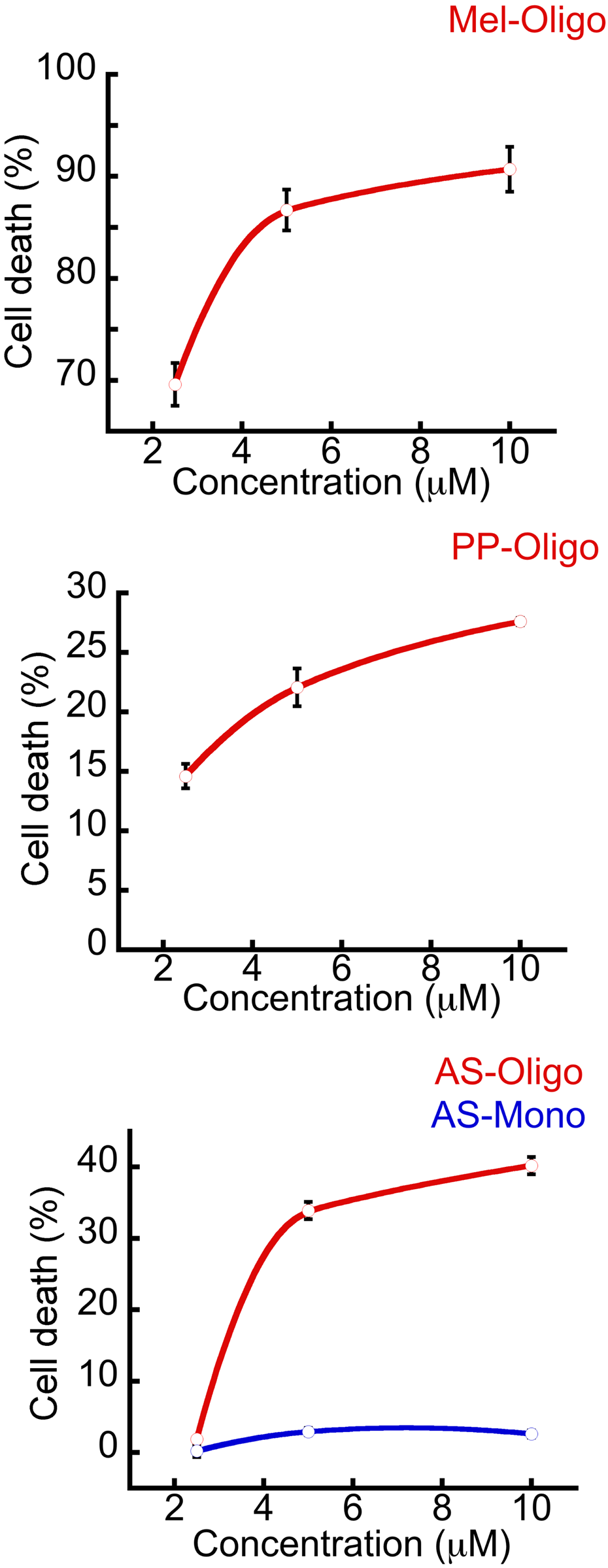

Supplement: S5 Fig — Different concentrations of oligomers (2.5 μM, 5.0 μM and 10 μM) were exposed to SH-SY5Y cells in cell culture for 30 h and then LDH assay was performed to quantify the cell death. Different concentrations of AS monomers were used as control. (TIF) [file pone.0120346.s005.tif]

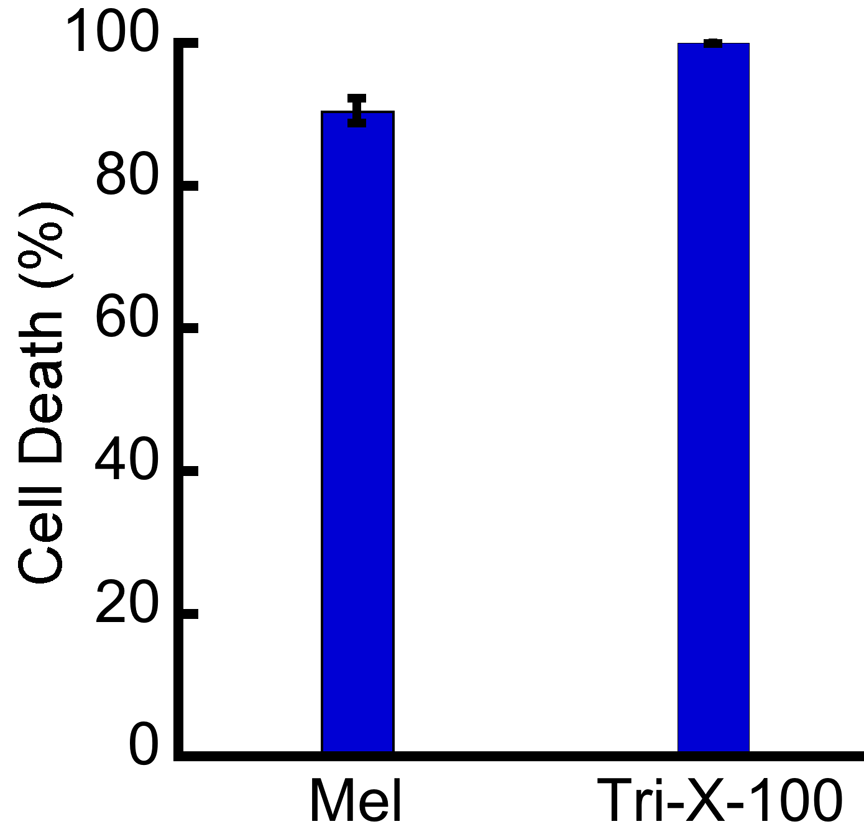

Supplement: S6 Fig — Cytotoxicity of freshly dissolved Mel (10 μM) was measured using LDH assay in SH-SY5Y cells. Triton-X-100 (0.5%) was used as positive control. (TIF) [file pone.0120346.s006.tif]

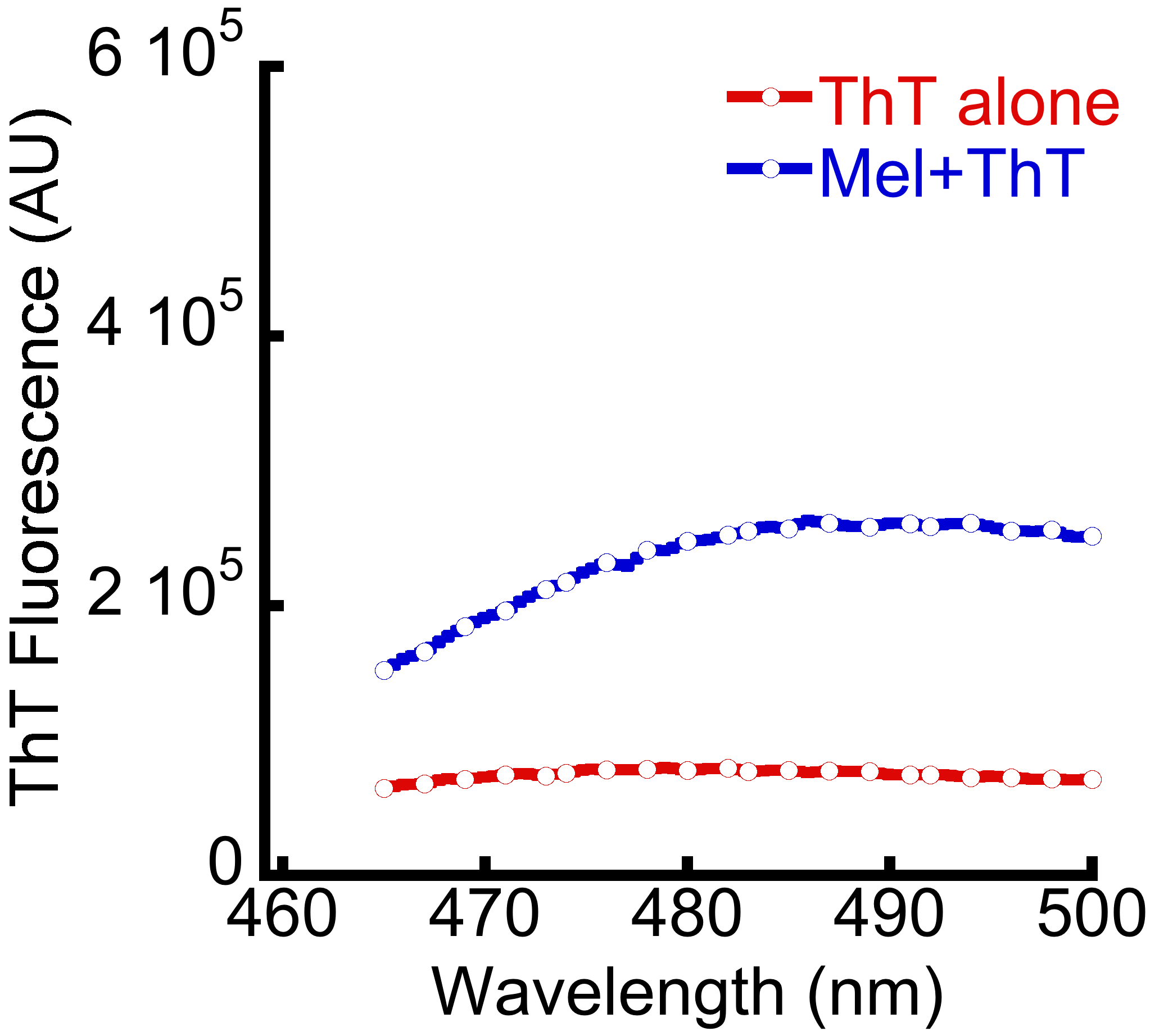

Supplement: S7 Fig — ThT fluorescence of Mel (day 0) after addition of SDS. SDS (2.5 mM) was added to Mel solution (25 μM) and then ThT fluorescence spectrum was recorded immediately after addition of ThT to this sample (d0). (TIF) [file pone.0120346.s007.tif]

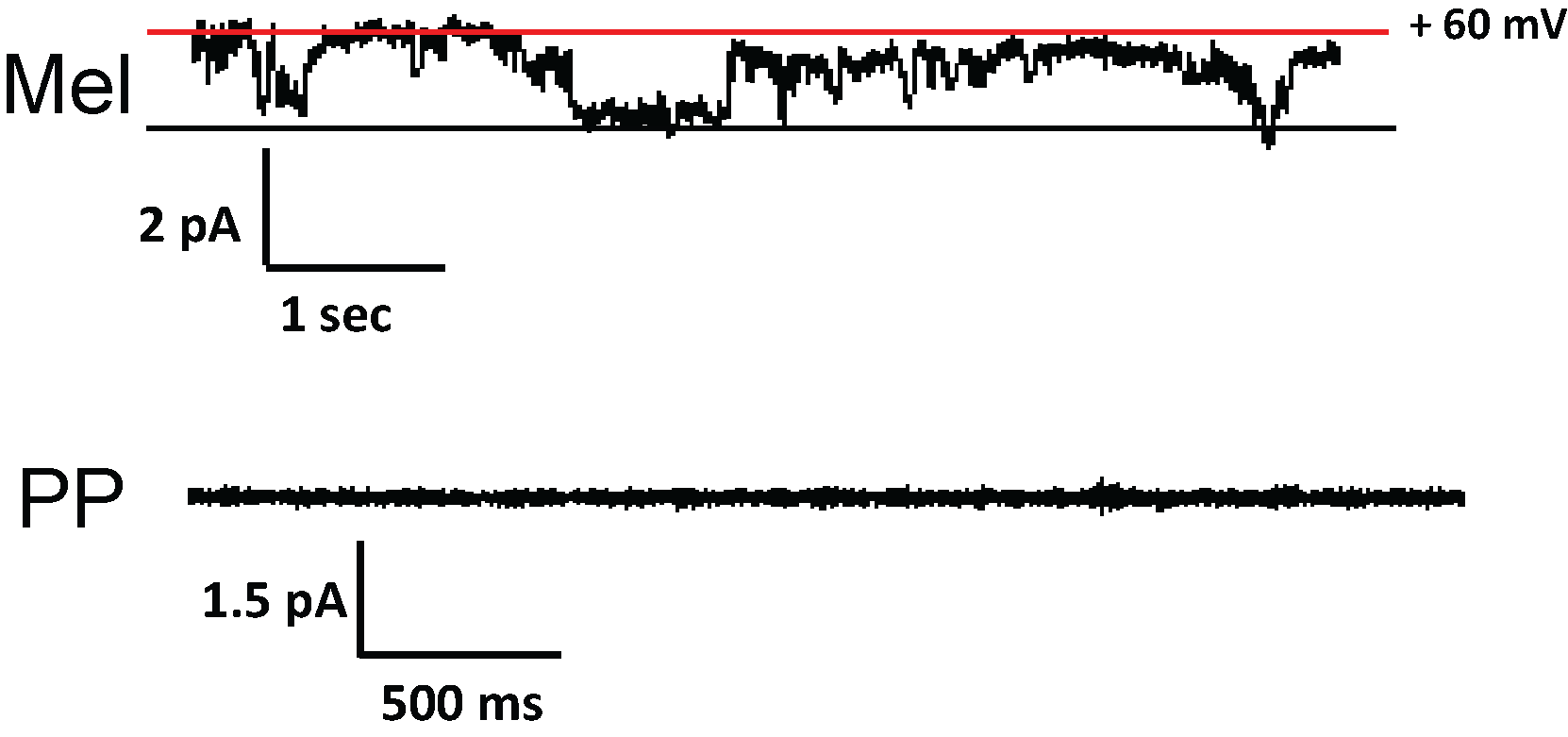

Supplement: S8 Fig — In the recording of single channel current, PP sample (incubated for two weeks in the absence of heparin) did not show any channel activity. However, Mel (incubated for two weeks in the absence of heparin) showed channel activity. (TIF) [file pone.0120346.s008.tif]
